# Systematic review: cardiovascular safety profile of 5-HT_4_ agonists developed for gastrointestinal disorders

**DOI:** 10.1111/j.1365-2036.2012.05011.x

**Published:** 2012-02-22

**Authors:** J Tack, M Camilleri, L Chang, W D Chey, J J Galligan, B E Lacy, S Müller-Lissner, E M M Quigley, J Schuurkes, J H Maeyer, V Stanghellini

**Affiliations:** * Department of Clinical and Experimental Medicine, University of LeuvenLeuven, Belgium; † Department of Gastroenterology and Hepatology, Mayo ClinicRochester, MN, USA; ‡ Department of Medicine, Division of Digestive Diseases, David Geffen School of Medicine at UCLACA, USA; § Department of Internal Medicine, University of Michigan Health SystemAnn Arbor, MI, USA; ¶ Department of Pharmacology and Toxicology, Michigan State UniversityEast Lansing, MI, USA; ** Dartmouth-Hitchcock Medical CenterLebanon, NH, USA; †† Department of Internal Medicine, Park-Klinik WeissenseeBerlin, Germany; ‡‡ Department of Medicine, Alimentary Pharmabiotic Centre, University College CorkCork, Ireland; §§ Shire-Movetis NVTurnhout, Belgium; ¶¶ Department of Clinical Medicine, University of BolognaBologna, Italy

## Abstract

**Background:**

The nonselective 5-HT_4_ receptor agonists, cisapride and tegaserod have been associated with cardiovascular adverse events (AEs).

**Aim:**

To perform a systematic review of the safety profile, particularly cardiovascular, of 5-HT_4_ agonists developed for gastrointestinal disorders, and a nonsystematic summary of their pharmacology and clinical efficacy.

**Methods:**

Articles reporting data on cisapride, clebopride, prucalopride, mosapride, renzapride, tegaserod, TD-5108 (velusetrag) and ATI-7505 (naronapride) were identified through a systematic search of the Cochrane Library, Medline, Embase and Toxfile. Abstracts from UEGW 2006–2008 and DDW 2008–2010 were searched for these drug names, and pharmaceutical companies approached to provide unpublished data.

**Results:**

Retrieved articles on pharmacokinetics, human pharmacodynamics and clinical data with these 5-HT_4_ agonists, are reviewed and summarised nonsystematically. Articles relating to cardiac safety and tolerability of these agents, including any relevant case reports, are reported systematically.

Two nonselective 5-HT_4_ agonists had reports of cardiovascular AEs: cisapride (QT prolongation) and tegaserod (ischaemia). Interactions with, respectively, the hERG cardiac potassium channel and 5-HT_1_ receptor subtypes have been suggested to account for these effects. No cardiovascular safety concerns were reported for the newer, selective 5-HT_4_ agonists prucalopride, velusetrag, naronapride, or for nonselective 5-HT_4_ agonists with no hERG or 5-HT_1_ affinity (renzapride, clebopride, mosapride).

**Conclusions:**

5-HT_4_ agonists for GI disorders differ in chemical structure and selectivity for 5-HT_4_ receptors. Selectivity for 5-HT_4_ over non-5-HT_4_ receptors may influence the agent's safety and overall risk–benefit profile. Based on available evidence, highly selective 5-HT_4_ agonists may offer improved safety to treat patients with impaired GI motility.

## Introduction

Disorders of gastrointestinal (GI) motility are considered a major pathophysiological mechanism underlying symptoms of functional GI disorders.^2008^ Therapeutic agents have been designed to stimulate muscle activity to address the underlying hypomotility associated with disorders such as slow-transit constipation, gastroparesis and ineffective oesophageal motility.^2008^ Activation of 5-HT_4_ receptors on cholinergic nerve endings in the enteric nervous system enhances the release of acetylcholine from motor neurons, thereby stimulating GI propulsive motility.^2^, ^3^ From these pharmacological observations, 5-HT_4_ receptor agonists have been developed for the treatment of hypomotility disorders. Nonselective 5-HT_4_ receptor agonists such as cisapride and tegaserod were successfully developed for the treatment of hypomotility disorders of the upper and lower GI tract, respectively.^4^, ^5^ Although both drugs saw broad clinical use, they were associated with cardiovascular adverse events (AEs).^6^, ^7^, ^8^ Cisapride was subsequently withdrawn from the global market in 2000 and, since 2009, tegaserod, which never received approval in the European Union (EU), has been limited to emergency use in the United States.^9^, ^10^ These cardiovascular AEs, which may be more related to a lack of selectivity of certain compounds or classes of compounds, rather than to genuine 5-HT_4_ receptor-mediated effects, have strongly impacted the perceived risk–benefit ratio of 5-HT_4_ receptor agonists. Meanwhile, a newer generation of selective 5-HT_4_ receptor agonists is being developed for the treatment of GI motility disorders. In this article, we review the safety profile of older and newer 5-HT_4_ receptor agonists developed for GI disorders, focusing on their cardiovascular risk profile.

## Pharmacology of 5-HT_4_ Receptor Agonists

### Structure of 5-HT_4_ receptors

5-HT_4_ receptors are heptahelical receptors, which primarily couple to the stimulatory protein G_s_ and activate the 3′,5′ cyclic adenosine monophosphate-dependent protein kinase A pathway.^11^, ^12^ Most of the 5-HT_4_ receptor splice variants are identical up to leucine 358, but their intracellular C-terminal tails differ.^1998^ The splice variants 5-HT_4(a)_ and 5-HT_4(b)_ have been found in all species studied thus far, with 5-HT_4(b)_ being the dominant splice variant in human tissues.^2001^ Additional splice variants have also been identified in human (h5-HT_4(c)_, h5-HT_4(d)_, h5-HT_4(g),_ h5-HT_4(i)_ and h5-HT_4(n)_), mouse (m5-HT_4(e)_ and m5-HT_4(f)_) and rat (r5-HT_4(c1)_ and r5-HT_4(e)_)^13^, ^15^, ^16^ and more recently in porcine tissue.^2008^ The physiological implication of the multitude of splice variants and their differential coupling to signal transduction cascades remains unclear.

Furthermore, several observations suggest that there is cell type-, tissue-specific or disease-state-specific expression (e.g. in gastroparesis) of certain splice variants.^13^, ^18^, ^19^, ^20^, ^21^ However, currently, there are no drugs which reliably discriminate among 5-HT_4_ receptor splice variants, but such drugs could provide an interesting alternative opportunity for tissue-specific drug targeting.

### Tissue distribution of 5-HT_4_ receptors

5-HT_4_ receptors are localised to neurons in the central nervous system, including the prefrontal cortex,^22^, ^23^ hippocampus^12^, ^22^ and mesolimbic and nigrostriatal dopamine systems.^2007^ Functional 5-HT_4_ receptors are also found in the GI tract, bladder and heart.^25^, ^26^, ^3^

In the GI tract, 5-HT_4_ receptors are expressed in enteric neurons^2008^ as well as smooth muscle cells.^28^, ^29^, ^30^ As a major consequence of 5-HT_4_ receptor activation, acetylcholine is released from interneurons and motor neurons, thus increasing propulsive motility.^30^–^37^

### Classes of 5-HT_4_ receptor agonists

Several different classes of 5-HT_4_ receptor agonists have been developed for the treatment of GI disorders. We review those classes here, focusing on their affinity and selectivity for the 5-HT_4_ receptor, as well as any tissue-dependent agonism (partial or full agonism) that might arise (in part) from differences in receptor reserve or coupling efficiency between different tissues.^2006^ Whether or not a drug is a full or partial agonist in a given tissue may depend on the receptor concentration or the efficiency of the receptor-effector coupling. Thus, it may be feasible to obtain a certain degree of tissue selectivity with a low efficacy agonist or low doses of a high efficacy agonist (in favour of tissues with high receptor reserve for the given agonist, such as the GI tract), as tissues with no receptor reserve for the agonist would not be stimulated by this drug.

#### Benzamides

**Figure 1:**
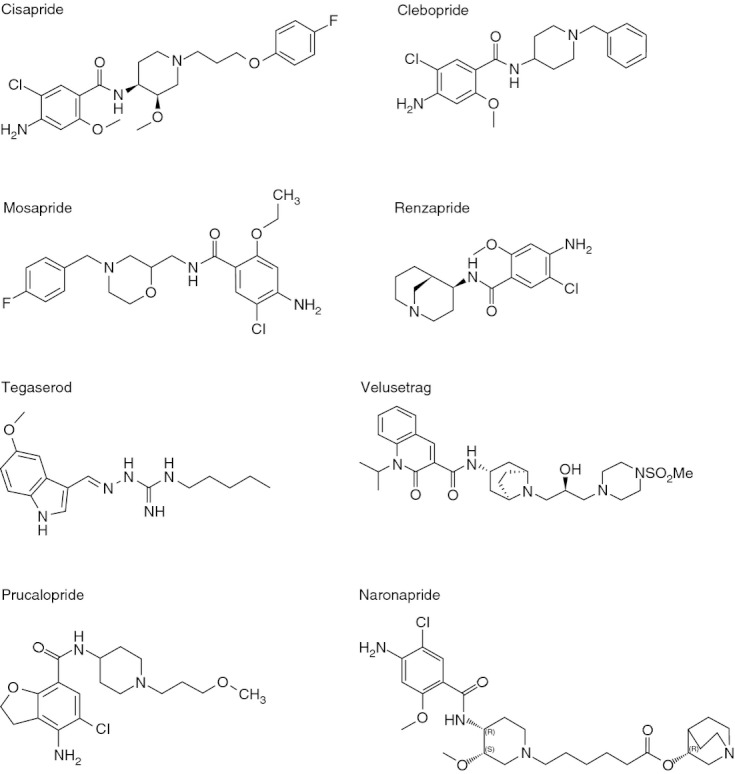
Molecular structure of 5-HT_4_ agonists.

The substituted benzamides, including metoclopramide, cisapride, renzapride, mosapride, clebopride and naronapride (ATI-7505) are 5-HT_4_ receptor agonists with moderate affinity and poor selectivity for the 5-HT_4_ receptor (Figure 1).^1995^ Metoclopramide is also an antagonist at D_2_ dopamine receptors and at 5-HT_3_ receptors, while cisapride blocks 5-HT_2_ and 5-HT_3_ receptors and the human ether-a-go-go-related gene (hERG)-encoded K^+^ channel.^39^, ^40^ The consequences of these interactions are clearer for some than for others; for example, the prolongation of cardiac action potential repolarisation and, thus, QT (the time elapsing from the beginning of the QRS complex to the end of the T wave in an electrocardiogram) interval, due to the blockade of the hERG channel by cisapride, is likely to underlie the arrhythmogenic potential of this nonselective 5-HT_4_ receptor agonist.^7^, ^41^ These non-5-HT_4_ receptor sites of action, coupled with their tissue-dependent pharmacodynamics, complicate the description of the actions of benzamides *in vivo*.

In the rat oesophagus, 5-HT_4_ receptors are localised to the muscularis mucosae (smooth muscle), where they mediate relaxation. In this tissue, cisapride, renzapride and mosapride have 80–90% of the intrinsic activity of 5-HT.^28^, ^42^ In the guinea-pig distal colon, cisapride, renzapride and mosapride mediate a contractile response and have 80–100% of the intrinsic activity of 5-HT.^1993^ Therefore, in both the guinea pig colon and rat oesophagus, benzamides are full agonists. In contrast, in the guinea pig ileum, cisapride, renzapride and mosapride have only 50–60% of the intrinsic activity of 5-HT.^42^, ^32^ Similarly, cisapride has <60% of the intrinsic activity of 5-HT in relaxing circular muscle strips of the canine rectum.^1999^

Little preclinical information is available on naronapride, another substituted benzamide 5-HT_4_ receptor agonist designed to have the same therapeutic benefit as cisapride, but without the side effects. Naronapride is structurally similar, but more selective, than cisapride, with minimal hERG channel activity as well as minimal-to-no activity at 5-HT_3_ receptors.^2007^ Naronapride stimulates GI motility *in vivo* in dogs^2004^ and in humans.^2007^

#### Carbazimidamides

Tegaserod is an indole carbazimidamide agonist with high affinity for the 5-HT_4_ receptor, but it is also a 5-HT_2(a)_ and 5-HT_2(b)_ receptor antagonist and 5-HT_1_ receptor agonist (Figure 1).^46^, ^47^, ^13^ The contribution of 5-HT_2B_ receptor antagonism to the therapeutic actions of tegaserod on gut motility remains unknown. Early studies showed that tegaserod facilitates the peristaltic reflex *in vitro* in human small intestine, and guinea pig and rat colonic preparations.^1998^ When agonist-evoked contractions of the guinea pig ileum were studied, tegaserod was equipotent with 5-HT, but had only 30% of the intrinsic activity of 5-HT.^49^, ^50^ *In vitro* studies comparing 5-HT_4_ receptor-mediated relaxation of canine rectal smooth muscle showed that tegaserod was 10-fold less potent than 5-HT and had only 55% of the intrinsic activity of 5-HT.^1999^ In the porcine stomach, tegaserod had approximately the same intrinsic activity as 5-HT when increases in cholinergic neurogenic contractions were measured.^2006^ These data indicate that tegaserod is a potent 5-HT_4_ receptor agonist in all tissues tested. While tegaserod is frequently identified as a partial receptor agonist, this property is tissue-dependent.^27^, ^38^

#### Benzofurancarboxamides

Prucalopride belongs to the class of benzofurancarboxamide agonists, which have high affinity and selectivity for the 5-HT_4_ receptor, and tissue-specific agonist activity (Figure 1).^27^, ^38^ The most pronounced effect of prucalopride is stimulation of colonic motility.^50–53^ Prucalopride is 10-fold less potent than 5-HT in causing relaxation of canine rectum circular muscle, with 82% of the intrinsic activity of 5-HT.^1999^ In guinea pig colon, prucalopride is approximately 10-fold less potent than 5-HT in stimulating peristalsis, but was equally effective as 5-HT (i.e. the maximal effect reached at high concentrations is similar to that of 5-HT) in this assay.^2001^ *In vivo*, prucalopride dose-dependently stimulated high-amplitude clustered contractions in the proximal colon and inhibited contractile activity in the distal colon.^2001^ Prucalopride also induced giant migrating contractions, the canine equivalent of mass movements.^2001^

#### Other agonists

Velusetrag (TD-5108) is a dihydroquinoline-carboxylic acid derivative that has high affinity and selectivity for 5-HT_4_ receptors in a number of *in vitro* assays, and is an effective stimulant of GI motility *in vivo* (Figure 1). Velusetrag has comparable intrinsic activity with 5-HT in the guinea pig colon and rat oesophagus tunica muscularis mucosa preparations, and has little or no activity at heterologously-expressed sodium channels or potassium channels.^2008^ Velusetrag is more potent than tegaserod, cisapride and mosapride at stimulating colonic transit in conscious guinea pigs, and approximately equipotent with tegaserod at stimulating small intestinal motility in conscious dogs or relaxing the oesophagus in anaesthetised rats.^2008^

Several other 5-HT_4_ receptor agonists have been developed for use in GI disorders, but development has ceased or limited data are available, so they will not be reviewed here. These agents include cinitapride (LAS17177), lintopride (STIL 2875), lirexapride (JL17454/CHF17454), PF885706 and E3620.

### Conclusions on pharmacology of 5-HT_4_ agonists

5-HT_4_ receptors are a widely expressed and dynamic class of receptors and 5-HT_4_ receptor agonists are effective stimulants of GI motility. Differences in the pharmacology of 5-HT_4_ receptor agonists originates mainly from differences in their selectivity for 5-HT_4_ over other receptor types, such as 5-HT_1_ and 5-HT_2_ receptors, and the hERG channel (Table 1), or by their degree of tissue-specific agonism.

**Table 1:** Receptor binding profile of 5-HT_4_ agonists for GI disorders, at therapeutic concentrations

	Receptor binding profile at therapeutic concentrations					
Drug	5-HT_4_	5-HT_3_	5-HT_2_	5-HT_1_	D_2_	hERG
Cisapride	+	+	+			+
Tegaserod	+	+	+	+		
Renzapride	+	+				
Clebopride	+	+			+	
Mosapride	+	+				
Prucalopride	+					
Velusetrag	+					
Naronapride	+					

GI, gastrointestinal.

+ indicates affinity for this receptor (as either agonist or antagonist) that is likely to be clinically relevant at concentrations necessary for 5-HT_4_ agonism (i.e. for therapeutic action). Information from De Maeyer *et al*.2008

## Methods

The purpose of this review was to comprehensively search and systematically review the evidence on the safety of 5-HT_4_ receptor agonists developed for GI disorders, with particular reference to cardiovascular safety. Literature on pharmacokinetics, human pharmacodynamics and clinical data was also collected systematically, but summarised nonsystematically and therefore not presented here in full.

An independent researcher at the Royal Society of Medicine Library Search Services performed a comprehensive search of the Cochrane Library (including the Cochrane Database of Systematic Reviews, the Database of Abstracts of Reviews of Effects, the Cochrane Central Register of Controlled Trials, the Health Technology Assessment Database and the NHS Economic Evaluation Database), Medline (1949 – April 2009), Embase (1974 – April 2009) and ToxFile (1900 – April 2009) in June 2009. The search encompassed all types of clinical trials as well as systematic reviews, meta-analyses and case reports, using the terms ‘cisapride’ OR ‘clebopride’ OR ‘prucalopride’ OR ‘mosapride’ OR ‘renzapride’ OR ‘tegaserod’ OR ‘TD-5108’ OR ‘ATI-7505’, including derivatives of these terms and other names used for these drugs. Limits applied were: English language, humans and adults. No date limitation was imposed. The searches were repeated in June 2010 to identify any new reports that emerged during the time taken to develop the manuscript. Upon revision of the manuscript in December 2011, an additional search for pharmacokinetic information was conducted and relevant references were obtained. Authors also contacted the medical information departments of relevant pharmaceutical companies to request further unpublished information or abstracts presented at conferences. The reference lists of retrieved articles were also reviewed.

Abstracts presented at UEGW 2006–2008 and at DDW 2008–2010 (i.e. for all years where abstracts were searchable electronically) were also searched by medical writing support staff, for mention of the drugs of interest (listed above), and those reporting data not already published in the retrieved articles were included in the analysis.

The Royal Society of Medicine use an automated duplicate checker, followed by hand-screening by the researcher to remove duplicates before records were reviewed. The remaining abstracts were filtered for relevance according to predefined eligibility criteria: studies had to relate to GI disorders, to the study drug (listed above), be either clinical studies or systematic reviews (including meta-analyses) and case reports were only included if they related to cardiac safety. Resulting abstracts were then hand-searched against the same eligibility criteria by a second reviewer, and relevant abstracts were segregated into pharmacokinetics, human pharmacodynamics, clinical studies and safety/tolerability, and full-text of these articles obtained. Full text was then reviewed independently by the authors. Cardiac-related safety/tolerability information was systematically reviewed and data retrieved from the full text by the authors were included in the manuscript, as the study authors had originally reported (without using any specific summary measures or additional analyses). Any discrepancies were discussed with the authors. No specific form was used to extract data, risk of bias of individual studies was not formally assessed and data from individual studies were not combined.

## Results

The literature searches, after removal of duplicates, returned a total of 1164 articles. A total of 61 conference abstracts were identified.

The original cisapride search returned 582 articles. Of these, 381 were selected for full-text review (20 pharmacokinetic, 141 pharmacodynamic, 183 clinical efficacy, 37 safety). Of these, 75 were considered relevant and cited in this article; 31 additional articles were identified and included. In the 2010 update to the search, four articles were identified, two of which met inclusion criteria. None of the three conference abstracts identified were considered relevant.

The tegaserod search returned 162 articles in the original search, and 13 in the update. Of these, 100 were selected for full-text review and 22 were considered relevant and cited in this article; nine additional articles were identified and included. Upon revision of the manuscript in 2011, one additional article was identified in the search and included in the manuscript. None of the 19 conference abstracts identified was included in this report.

The renzapride search returned 105 articles in the original search and two in the update. Of these, 17 were selected for full-text review and seven were considered relevant and cited in this article; two additional articles were identified and included. Upon revision of the manuscript in 2011, one additional article was identified in the search and included in the manuscript. No renzapride conference abstracts were identified.

The clebopride search returned 58 results, of which 38 were selected for full-text review. Of these, eight were included in this report (two of these were originally excluded for not meeting inclusion criteria, but provided useful supporting information so were included by the author). Of the 16 safety-related articles selected for full-text review, none related to cardiac safety; two representative articles (movement disorders) were included in this report. Four additional articles, as well as the clebopride prescribing information and an *in vitro* report were identified and included. Upon revision of the manuscript in 2011, three additional articles were identified in the search and included in the manuscript. No clebopride conference abstracts were identified.

The original mosapride search identified 120 results, and the second search an additional four items. Of these, 41 were selected for full-text review (including three articles relating to safety). Sixteen were selected to report here, including the two relevant safety reports. An additional seven reports were identified through other sources and included, as well as six background articles (including the mosapride prescribing information). Upon revision of the manuscript in 2011, one additional article was identified in the search and included in the manuscript. None of the 17 mosapride conference abstracts identified were included in this report.

The prucalopride search returned 121 articles in the original search, and seven in the update. Of these, 32 were selected for full-text review and 16 considered relevant and cited in this article; four additional articles were identified and included. Of the 20 conference abstracts identified, five presented relevant information from studies not already covered by full publications, so were included in this report. One related to cardiac safety.

The ATI-7505 search returned 11 articles in the original search and one in the update. Of these, one was selected for full-text review and one considered relevant and cited in this article; four additional articles were identified and included. Upon revision of the manuscript in 2011, one additional article was identified in the search and included in the manuscript. No conference abstracts were identified.

The TDI-5108 search returned five articles in the original search and three in the update. Of these, three were selected for full-text review and two considered relevant and cited in this article; two additional articles were identified and included. Two conference abstracts were identified and included in this report.

### Nonselective 5-HT_4_ receptor agonists

Historically, metoclopramide was the first 5-HT_4_ receptor agonist to be used for the treatment of hypomotility disorders. The drug is also a D_2_ receptor antagonist, an action which underlies the well-established, potentially irreversible, neurological side effects that may occur with metoclopramide intake,^2010^ and therefore will not be described in detail.

#### Cisapride

Cisapride, an agonist at 5-HT_4_ receptors and an antagonist at 5-HT_3_ and 5-HT_2_ receptors, was introduced worldwide in the 1990s, and has widespread prokinetic effects throughout the GI tract. Although originally employed in a wide range of motility disorders, cisapride was approved for the treatment of acute and severe exacerbations of demonstrated chronic idiopathic or diabetic gastroparesis after failure of other treatment options in the EU, and was approved by the U.S. Food and Drug Administration (FDA) for nocturnal heartburn only.

#### Pharmacokinetics and pharmacodynamics

The pharmacokinetics and pharmacodynamics of cisapride are summarised in Tables 2 and 3 respectively.

**Table 2:** Pharmacokinetics of 5-HT_4_ agonists

5-HT_4_ receptor agonist	Bioavailability	Plasma levels (*t*_max_)	Protein binding	T_1/2_	Metabolism and elimination (hepatic)	Metabolism and elimination (renal)
Nonselective 5-HT_4_ receptor agonists						
Cisapride	∼40–50% (absolute)^2002^	1–2 h^1998^	∼98%^1998^	∼10 h^2002^ (potentially prolonged in patients with hepatic impairment/elderly)^1998^	P450 enzyme system^2002^	Unchanged renal or faecal recovery are <10% of the ingested dose following oral administration
Tegaserod	11 ± 3% (absolute)^1999^	1.3 h^1999^	∼98%[Bibr b144]	11 ± 5 h (terminal)^1999^	*In vitro* studies suggest that CYP-mediated metabolism plays an insignificant role in the elimination of tegaserod^2001^	Transported by P-glycoprotein efflux pump[Bibr b146]2/3 excreted unchanged in faeces; 1/3 excreted in urine as main metabolite[Bibr b144] after acid-catalysed hydrolysis in the stomach and glucuronidation
Renzapride	Good^2008^	∼1.4 h^2008^		∼6 h (plasma)^2008^	Not metabolised through major CYP drug-metabolising enzymes and does not inhibit CYP450-mediated metabolism of other drugs^2008^	Renal excretion^2006^; eliminated unchanged in the urine^2008^
Clebopride	Good^1991^	1.5–1.6 h[Bibr b149]		36.5 (0.5 mg) and 26.5 (1 mg) h (serum)[Bibr b149]	CYP3A4 metabolisation^2011^ Excreted in faeces (maximal excretion 2–3 h after ingestion)^1991^	About 50% renal excretion^1990^
Mosapride	8% (in dogs) and 14% (in monkeys; oral)^1993^	0.5–1.0 h^1993^			Hepatic metabolism^2003^; first-pass in the liver (principal metabolite M1), excreted in both urine and faeces^53^, ^54^	Elimination via urine and faeces^2011^
Selective 5-HT_4_ receptor agonists						
Prucalopride	>90% (oral)[Bibr b156]	2.1 ± 0.9 h	28–33%	21.2 ± 3.7 h (terminal)	No hepatic metabolisation At least 6% excreted unchanged in faeces[Bibr b156]	∼60% excreted unchanged in urine[Bibr b156]
Velusetrag	∼30% (estimate; oral)	4–6 h		∼13 h	Hepatic metabolisation, substrate of CYP3A4 and P-glycoprotein^2008^	Major metabolite (THRX-830449) has equivalent potency to parent; elimination *t*_1/2_ 16 h (single dose) & 35 h (multiple dose)[Bibr b135]
Naronapride					No CYP450 metabolisation^2007^	Excretion in faeces (one third, unchanged) and urine (metabolite)^2011^

**Table 3:** Pharmacodynamics of 5-HT_4_ agonists

	Cisapride	Tegaserod	Renzapride	Clebopride	Mosapride	Prucalopride	Velusetrag	Naronapride
Oesophagus								
Salivary flow	↑ in GERD^158–168^ Amplitude +/− in HV^160^, ^169^, ^170^							
Oesophageal peristalsis/amplitude	↑ in GERD^158–170^	–^171^, ^172^		↑^1989^	↑ in GERD^2003^			
Oesophageal emptying	↑ in GERD^158–168^ Amplitude +/− in HV^160^, ^169^, ^170^							
Oesophageal acid clearance	↑ in GERD^158–168^ Amplitude +/− in HV^160^, ^169^, ^170^							
Oesophageal acid exposure time					Reduced in GERD^1998^			
No. of reflux events, and no. >5min					Reduced in GERD^1998^			
LES pressure	↑ in HV^161^, ^169^, ^170^, ^179^ ↑or – in GERD^160-162^, ^168^, ^171^				– in HV^2006^			
LES tone				↑^173^, ^179^				
No. of TLESRs	+/− in HV^177^, ^180^				– in HV^2006^			
Stomach & duodenum								
Post-prandial acid secretion/gastric acidity	+/− in GERD^165^, ^177^, ^181^, ^182^							
Electrogastrographic patterns	Improved in FD^2000^ ^1998^							
Gastric tone	↑ in GP^1999^							
Gastric emptying time	↓ in HV^186^, ^187^, ^188^, ^189^ and patients^164^, ^185^, ^188^, ^190^, ^191^, ^192^, ^193^, ^194^, ^195^, ^196^, ^197^, ^198^, ^199^, but inconsistent^200^, ^201^, ^202^	↓ in HV^2005^	↓ in DGp (solid and liquid)^1991^	↓[Bibr b102]	Improved^2007^ in HV^1991^, DPAN^1992^, PD^2005^	– in HV^1999^	↓ in HV^2010^	↓ in HV^2007^
Amplitude of gastric contractions								
Gastric compliance/gastric accommodation	+/− in HV^208^, ^209^	↑ in FD and HV^2007^						
Gastric transit time						↓ in C^2001^		
Gastro–duodenal transit time				↓ in Gp^1982^				
Relaxation of pyloric sphincter								
Antroduodenal motility	↑ in HV^188^, ^209^, ^213^, and patients^197^, ^200^, ^214^, ^215^, ^216^, but inconsistent^182^, ^185^, ^188^							
Duodenal and jejunal contractions								
Gall bladder emptying	↑ in HV^217^, ^218^							
Stomach & intestines								
Gastrointestinal transit time			↓ in IBS-C^2006^					
Small & large intestine								
Small intestine motility	↑ in HV^219^, ^220^, ^221^, ^222^ and IBS^220^, ^223^							
Small intestine transit time	– in HV,^1987^ ↓ in CIIP^1986^	↓ in HV^2005^						
Colonic filling							↑ in HV^2010^	
Ascending colon emptying							↓ in HV^2010^	
Colonic transit time	↓ in HV^1987^ and constipation^226^, ^227^, ^228^, ^229^	↓ in HV and IBS-C^171^, ^172^	↓ in IBS-C^96^, ^99^			↓ in HV^1999^ and C^2001^	↓ in HV^2010^	↓ in HV^2007^
Colonic motor activity	↑ after resection^1995^							
Anorectal function						– in HV^051^, ^231^ and C^2002^		
Anorectal sensation		↑ in HV^2003^				– in HV^051^, ^231^ and C^2002^Heightened in C (mechanical and electrical)^2002^		
Stools								
Stool consistency							↓ in HV^2010^	↓ in HV^2007^
Bowel frequency							↑ in HV^2010^	
Whole gut transit						↓ in HV^1999^ and C^2001^		

↑, increased; ↓, decreased; –, no effect; +/−, mixed effects; AN, anorexia nervosa; C, constipation; CI, critical illness; CIIP, chronic idiopathic intestinal pseudo-obstruction; DPAN, diabetic patients with autonomic neuropathy; DGp, diabetic gastroparesis; FD, functional dyspepsia; Gp, gastroparesis; HV, healthy volunteers; IBS-C, constipation-predominant irritable bowel syndrome; LES, lower oesophageal sphincter; PD, Parkinson's disease; TLESR, transient lower oesophageal sphincter relaxations.

#### Clinical trials

Clinical trials, summarised in Supplementary Table S1, have explored the efficacy of cisapride in a number of GI conditions, including GERD, functional dyspepsia, gastroparesis, chronic intestinal pseudo-obstruction, post-operative ileus and chronic constipation, but evidence of an effect was not robust enough to obtain regulatory approval for indications other than nocturnal heartburn. In 2000, cisapride was withdrawn from the market worldwide, due to concerns over cardiovascular safety (see safety and tolerability section).

#### Safety and tolerability

Initial experience with cisapride, including post-marketing data from two study populations totalling over 23 000 patients, suggested that the drug was remarkably safe, with diarrhoea (incidence 4.1%), abdominal pain (1.6%), nausea or vomiting (1.5%), headache (1.4%) and constipation (1.2%) being the most frequently reported AEs.^1997^ In an epidemiological study of almost 37 000 patients prescribed cisapride in the UK and Canada, serious cardiac rhythm disorders were not found to be inordinately associated with cisapride use; however, the 95% confidence interval was large (0.2–9.8), such that an increase in risk could not be ruled out definitively.^1999^ In children without underlying cardiac disease or electrolyte imbalance, cisapride was found to have no significant effect on cardiac electrical function,^2001^ and a meta-analysis of randomised, controlled clinical trials among children with GERD found that although there was no evidence of adverse or harmful events, there were no significant clinical benefits.^2003^

Reports of cardiac events began to accumulate, including palpitations^1992^ and instances of an unusual tachyarrhythmia, torsades de pointes, as well as ventricular fibrillation and sudden death started to appear.^2000^ It soon came to be recognised that cisapride use could result in prolongation of the QT interval and, thereby, increase the risk of arrhythmia, a phenomenon well described in association with a number of other drugs, most notably quinidine, but including procainamide, sotalol, amiodarone, disopyramide, macrolide antibiotics (including erythromycin), astemizole, terfenadine, phenothiazines and tricyclic antidepressants. An increased risk was present when cisapride levels were higher, e.g. through concomitant use of CYP450 inhibitors. Indeed, the majority of cardiac AEs occurred when cisapride was used in patients with other risk factors, which included co-administration with other drugs (e.g. triazole antifungals and retrovirals) and foods such as grapefruit juice,^1999^ which inhibit hepatic cytochrome (CYP) P450 3A4 resulting in high plasma levels of cisapride, or that also caused QT prolongation, as listed above. It also became clear that the likelihood of arrhythmia in association with cisapride use was greater among those with serious underlying disease states such as heart disease, heart failure, respiratory failure, renal failure, hypokalaemia or hypomagnesaemia.

Subsequently, the cellular and molecular basis for the arrhythmogenic potential of cisapride was revealed. *In vitro* studies found cisapride to be a potent and dose-dependent blocker of the hERG channel,^1997^ which is the main channel responsible for the repolarisation phase of the cardiac action potential,^1998^ such that hERG blockade (or channel mutations) prolong the duration of the action potential by delaying the repolarisation phase.^2008^ Other studies suggested that it was not QT prolongation *per se*, but rather, the increase in dispersion of repolarisation that usually accompanies QT prolongation, which provides the arrhythmogenic substrate.^2003^

Although some studies suggested that cisapride had a low arrhythmogenic potential among neonates regardless of gestational age,^2002^ others drew attention to the low levels of CYP 3A4 in the neonatal liver^2001^ and the consequent effects on cisapride metabolism. Indeed, a pharmacokinetic study in premature infants demonstrated increased serum concentrations of cisapride and parallel prolongations of the QT interval.^2003^

Unfortunately, around the same time, the prokinetic properties of erythromycin had begun to be widely appreciated and its co-administration with cisapride, or the sequential use of i.v. erythromycin and oral cisapride, was not uncommon; thus, increasing the potential for QT prolongation, through its own effects on the QT interval as well as through its inhibition of CYP450.^070^, ^071^, ^072^ Others at risk were those with congenital prolongation of the QT interval, a family history of the long QT syndrome or those with significant bradycardia.

As large surveys in adults, children and neonates had indicated that such events were rare^058^, ^073^, ^074^, ^075^, ^076^ and that risks could be managed by an appropriate awareness programme, the initial response was a risk management programme identifying those at risk, as well as drugs that should not be co-administered with cisapride. Accordingly, in 1995, a ‘black box’ warning contraindicating the use of cisapride among those taking drugs that affected its metabolism was issued by the FDA. At that time, 34 cases of torsades de pointes, 23 of prolonged QT interval and 4 deaths had been reported.^2000^ In 1996, further warnings were issued in relation to concomitant medications that also prolonged the QT interval and in conditions that predisposed to cardiac arrhythmias. As a result, the ‘black box’ was expanded in 1998. However, the subsequent realisation that serious cardiac AEs could occur among low-risk groups, including children, coupled with the documentation of continued cisapride use in contraindicated situations^2001^ led to the commercial, worldwide withdrawal of the drug in July 2000.

#### Tegaserod

Tegaserod is a 5-HT_4_ receptor agonist with a high affinity for 5-HT_4_ receptors, but also relevant affinity for 5-HT_2(a/b)_, and 5-HT_1(a/b/d)_ receptors.^27^, ^77^ Tegaserod was approved in many countries for the treatment of IBS-C and chronic idiopathic constipation. In March 2007, tegaserod was withdrawn from most markets owing to an increased risk of cardiovascular AEs. It was reintroduced in the USA in July 2007 under a treatment investigational new drug protocol for IBS-C and chronic idiopathic constipation in women younger than 55 who are not at risk for certain cardiovascular events. Tegaserod was not approved for use in the EU as the Committee for Medicinal Products for Human Use was of the opinion that the benefit of tegaserod treatment did not outweigh its risks.

#### Pharmacokinetics and pharmacodynamics

The pharmacokinetics and pharmacodynamics of tegaserod are summarised in Tables 2 and 3 respectively.

#### Clinical studies

While the benefit of tegaserod therapy in patients with IBS-C has been fairly well demonstrated, evidence of efficacy in chronic idiopathic constipation and functional dyspepsia has been less convincing (summarised in Supplementary Table S2).

#### Safety and tolerability

The frequency of reported AEs in the 3-month trials with tegaserod varies considerably (e.g. 50–60% of US patients reported AEs),^2006^ whereas only 4% of the patients in an Asia-Pacific trial^2007^ and 38% (13% drug-related) in a European trial did so.^2005^ As this can be explained neither by different doses nor by trial design, cultural differences in the willingness to complain are the most likely explanation.

The most common AEs were diarrhoea, headache and abdominal pain,^078^, ^079^, ^080^, ^081^ and were graded as mild, sometimes as moderate.^2005^ Only diarrhoea was reproducibly more prevalent in the tegaserod than in the placebo groups, but this was not dose-related.^2006^ Diarrhoea occurred mainly in the first week of treatment and was often transient, resolving with continued treatment.^2006^ The safety profile did not change over a 12-month period with open treatment^2006^: headache became the most prevalent AE (10–15%); all other AEs, in particular diarrhoea, ranged below 10%.

Three AEs potentially attributable to tegaserod have gained particular attention, namely an increased rate of abdominal surgery, ischaemic colitis and cardiac events. They occurred so infrequently that they are unlikely to be identified in Phase III trials and may only come up in pooled analyses or during post-marketing surveillance, but they are severe enough to warrant concerns.^2004^ Regarding abdominal surgery in patients with IBS-C, a meta-analysis of 13 controlled trials was conducted, which found no significant difference in the rates of abdominal and pelvic surgery between patients receiving tegaserod (0.44%) and those on placebo (0.41%).^2004^

Patients with IBS have a higher risk of developing colonic ischaemia than the general population.^2004^ No cases of ischaemic colitis occurred in over 11 600 patients participating in clinical trials with tegaserod.^2004^ Although post-marketing reports have noted cases of ischaemic colitis^86^, ^87^ and intestinal ischaemia^2004^ in patients taking tegaserod, the incidence appears to be similar to the general population and is less than estimates for the IBS patient population.^2004^ Moreover, no mechanism has been identified through which tegaserod might predispose to ischaemic colitis.

The pre-marketing data of systematic cardiac safety assessment may be summarised as follows: in three randomised, double-blind, controlled, parallel-group clinical studies with more than 2500 IBS patients, prolongation of the QTc interval was similarly frequent between groups, as was the frequency of overall electrocardiographic abnormalities. No ventricular or supraventricular tachycardia was observed. In healthy volunteers, tegaserod at i.v. doses resulting in plasma concentrations up to 100 times those measured after therapeutic doses (6 mg b.d.) did not influence electrocardiographic variables.^2002^

Data collected from over 18 000 patients demonstrated cardiovascular AEs (myocardial infarction, unstable angina pectoris, stroke and one sudden death) in 13 of 11 614 patients treated with tegaserod (a rate of 0.11%) compared with 1 of 7031 patients treated with placebo (a rate of 0.01%). Therefore, in 2007, the FDA requested withdrawal from the market, citing a relationship between prescriptions of the drug and increased risks of heart attack or stroke. However, this was denied by the manufacturer, as all affected patients were said to have pre-existing cardiovascular disease or risk factors for such.[Bibr b6] Thus, no causal relationship between tegaserod use and cardiovascular events had been demonstrated. Indeed, a matched case–control study of tegaserod-treated patients with untreated patients found no association between tegaserod and adverse cardiovascular outcomes.^2009^

A hypothetical mechanism for tegaserod-related cardiac events was proposed involving interaction at 5-HT_1(b/d)_ receptors on coronary arterioles.^2004^ However, as tegaserod does not behave as a 5-HT_1(b)_ receptor agonist in a recent study of human isolated proximal and distal coronary arterioles, the mechanisms involved remain unclear.^2009^

#### Renzapride

Renzapride, a 5-HT_4_ receptor agonist and a 5-HT_3_ receptor antagonist,^2008^ has been evaluated in the treatment of IBS-C, but has not been approved in any part of the world.

#### Pharmacokinetics and pharmacodynamics

The pharmacokinetics and pharmacodynamics of renzapride are summarised in Tables 2 and 3 respectively.

#### Clinical studies

Clinical studies with renzapride have focused mainly on IBS (summarised in Supplementary Table S3). However, therapeutic margins were only minimal (5–6% over placebo), and this led to the discontinuation of the drug development programme.[Bibr b95]

#### Safety and tolerability

Renzapride was well tolerated; most AEs were mild-to-moderate in intensity and equally distributed across active or placebo treatment groups. The most common AEs were GI, with diarrhoea and abdominal pain being the most commonly associated with renzapride treatment.^096^, ^097^, ^098^, ^099^

*In vitro* cardiac conductivity studies in isolated Purkinje fibres showed no significant QT prolongation by renzapride. In transfected cells, renzapride was a 1000-fold less potent inhibitor of the hERG channel compared with cisapride.^2007^ In the clinical trial programme, no significant ECG alterations were observed (in particular no evidence of prolongation of the QT interval).^095^, ^096^, ^097^, ^098^, ^099^, ^101^

#### Clebopride

Clebopride is a D_2_ receptor antagonist, as well as a 5-HT_4_ receptor agonist and a 5-HT_3_ receptor antagonist.[Bibr b102] Clebopride is available in Italy and Spain as a gastroprokinetic drug.

#### Pharmacokinetics and pharmacodynamics

The pharmacokinetics and pharmacodynamics of clebopride are summarised in Tables 2 and 3 respectively.

#### Clinical studies

To our knowledge, the effects of clebopride on lower GI motility and therapeutic potential in lower GI indications have not been explored. Controlled studies investigating the effects of clebopride have primarily focused on FD, but are dated and are generally underpowered with methodological flaws compared with current requirements of good clinical practice (summarised in Supplementary Table S4).

#### Safety and tolerability

Antagonism of dopamine receptors in the GI tract promotes coordinated motor activity and accelerates transit, while blockade of D_2_ receptors in the area postrema exerts an anti-nausea, anti-emetic effect.^103^, ^104^ However, central D_2_ receptor blockade is also responsible for AEs, including extrapyramidal dystonic reactions and hyperprolactinaemia.^2004^ Comparative studies among prokinetics have demonstrated that clebopride is most associated with dystonic reactions^106^, ^107^ that are not limited to reversible Parkinsonian-like symptoms, but also include tardive, potentially irreversible, dyskinesia.^1993^ The calculated prevalence of movement disorders associated with chronic use of other antidopaminergics is around 1%,^1982^ but it is 4-fold higher for clebopride.^1996^ Conversely, clebopride exerts a less pronounced hyperprolactinaemic effect compared with any other antidopaminergic drug.^2004^

Substituted benzamides have been associated with dose-dependent cardiac AEs. Thus, although overt cardiotoxicity has not been reported in clinical studies on clebopride, the effects of the drug on cardiac action potential duration, hERG channel, and sodium channel currents were investigated *in vitro*.^2007^ Clebopride (10 μm) prolonged the action potential duration at 90% (but not 50%) repolarisation. Furthermore, an IC_50_ value of 0.62 ± 0.30 μm for hERG channel currents was determined. No effect was observed on sodium channel currents.^2007^ It was concluded that clebopride is sufficiently safe at therapeutic doses, but overdosing or impaired metabolism might be associated with torsadogenic effects.

#### Mosapride

Mosapride is primarily a selective 5-HT_4_ receptor agonist in the GI tract.^112^, ^113^, ^114^ Its principal metabolite is approximately 50% as potent as the parent compound at stimulating gastric motility, however, it is also a potent 5-HT_3_ receptor antagonist.^1993^ Mosapride is available as a prokinetic agent in a number of Asian countries.

#### Pharmacokinetics and pharmacodynamics

The pharmacokinetics and pharmacodynamics of mosapride are summarised in Tables 2 and 3 respectively.

#### Clinical trials

Several studies have been performed to assess the efficacy of mosapride for the treatment of FD. However, most studies were small and lack controls and, as such, failed to show significant symptomatic improvements (summarised in Supplementary Table S5).^2002^

#### Safety and tolerability

In contrast to cisapride, mosapride does not appear to have any significant effect on K^+^ channels. Using isolated rabbit Purkinje fibres and ventricular muscle, mosapride had little effect on the rapid component of the delayed rectifying K^+^ channels and no effect in hERG transfected cells.^039^, ^117^, ^118^ Using a rabbit model of the acquired long-QT syndrome, cisapride prolonged the QT interval, while mosapride did not,^1997^ despite its metabolism by CYP3A4.^2000^ In a 14-day study of mosapride (15 mg/day) in 10 healthy male volunteers, no ECG changes were noted despite co-administration of erythromycin.^2003^ In a separate study of 20 healthy volunteers who received a single dose of mosapride (10 mg), pulse, heart rate, QT interval and ECGs were no different after drug administration.^2002^ Furthermore, in a study of 18 patients who were taking a variety of psychiatric medications, co-administration of mosapride did not change any ECG parameters.^2000^ However, a case report described a 68-year-old man with sick sinus syndrome, requiring a permanent pacemaker and concomitant flecainide therapy, who developed a prolonged QTc interval after starting mosapride.^2001^ In summary, the studies published to date demonstrate that mosapride is safe without any significant cardiovascular effects.

### Selective 5-HT_4_ receptor agonists

#### Prucalopride

Prucalopride is a dihydrobenzofurancarboxamide derivative with distinct structural differences from other 5-HT_4_ receptor agonists such as cisapride and tegaserod. These differences are likely to account for the greater selectivity for the 5-HT_4_ receptor observed with prucalopride (>150× for prucalopride vs. <1 for cisapride and tegaserod).^1997^ Prucalopride was recently approved, on 15 October 2009, by the European Medicines Agency (EMA) for the symptomatic treatment of chronic constipation in women in whom laxatives fail to provide adequate relief.

#### Pharmacokinetics and pharmacodynamics

The pharmacokinetics and pharmacodynamics of prucalopride are summarised in Tables 2 and 3 respectively.

#### Clinical trials

The majority of clinical studies with prucalopride were conducted in patients with chronic constipation (summarised in Supplementary Table S6). Multiple randomised, controlled trials found that prucalopride at doses of 1–4 mg (q.d.) improved symptoms of chronic constipation, including stool frequency, stool consistency, straining and quality of life.^125^, ^126^, ^127^, ^128^

To date, no studies addressing the efficacy of prucalopride in the IBS-C or upper GI disorders, such as gastroparesis or FD, have been presented.

#### Safety and tolerability

Safety assessments were performed as part of the Phase III studies.^125^, ^127^, ^128^ Treatment-associated AEs were reported in 70–80% of patients randomised to placebo, 2 or 4 mg of prucalopride. The most common treatment-associated AEs included headache in 25–30% of prucalopride subjects vs. 12–17% of placebo; nausea (12–24% vs. 8–14%); abdominal pain (16–23% vs. 11–19%); and diarrhoea (12–19% vs. 3–5%). The majority of AEs occurred within the first 24 h of treatment and proved transient.

Pooled results of the three Phase III studies show that serious AEs (SAEs) were reported in 2.7% of patients receiving the recommended dose of 2 mg (*n* = 661) vs. 2.0% of patients receiving placebo (*n* = 659).^2008^ Discontinuation rates from AEs ranged from 4% to 15% among patients receiving prucalopride and from 2% to 7% among patients receiving placebo. Many discontinuations were the consequence of headache, abdominal pain, nausea, vomiting or diarrhoea which occurred on the first day of study drug administration.

Data from an open-label, long-term extension trial were recently reported.^2009^ In this study, 1775 constipated patients who had completed one of the Phase III studies were followed up for a mean of 231 days (range 1–721 days). Similar to the Phase III trials, the most common AEs were headache (31%), abdominal pain (24%), diarrhoea (20%), flatulence (16%) and nausea (15%). The most common AE rated as ‘severe’ was surgical intervention (3.3%). SAEs were uncommon (<0.5%). The most common AEs leading to discontinuation were abdominal pain (1.5%) and headache (1.5%). Two deaths occurred; one was deemed unrelated to prucalopride and no treatment information was available for the other.^2009^

Unlike other 5-HT_4_ receptor agonists, prucalopride has not been found to interact with the hERG channel or 5-HT_1(b)_ receptors, each postulated to be responsible for the development of adverse cardiovascular effects with other 5-HT_4_ receptor agonists.^39^, ^92^ Cardiovascular safety was evaluated in two Phase I, double-blind, controlled, two-way cross-over studies which included 32 and 24 healthy volunteers, in which prucalopride was escalated to a maximum dose of either 10 or 20 mg.^2009^ No clinically relevant differences in blood pressure or incidence of prolonged QTc were identified between groups. Similarly, no correlation was found between observed shifts in ECG parameters based upon prucalopride concentrations up to 10 times the recommended therapeutic dose. A small, transient increase in mean heart rate and associated decrease in the PQ and QT intervals was observed with prucalopride. Within-subject differences between the groups were not statistically different. Cardiovascular safety was also carefully assessed in the Phase III clinical trials. There were no differences in vital signs or ECG parameters between study participants randomised to placebo or either dose of prucalopride. The incidence of QT interval prolongation (>470 ms) was low (≤2.1%) and similar between groups.^125^, ^127^, ^128^ A single cardiovascular event, an episode of supraventricular tachycardia, occurred in a patient with a history of mitral valve prolapse and cardiac arrhythmias, who was randomised to prucalopride 2 mg.^2008^

Cardiovascular safety was also assessed as part of a study conducted in a high-risk population. For this study, 89 elderly patients residing in a nursing facility (mean age 83 years) were randomised to prucalopride (0.5, 1 or 2 mg) or placebo. Of the participants, 80% had prior history of cardiovascular disease. There were no differences in vital signs, laboratory results or ECG parameters between groups. Moreover, there were no differences in the incidence of prolonged QTc interval between groups.^2009^

#### Velusetrag (TD-5108)

Velusetrag is a high-affinity 5-HT_4_ receptor agonist, which is being developed for the treatment of chronic constipation.

#### Pharmacokinetics and pharmacodynamics

The pharmacokinetics and pharmacodynamics of velusetrag are summarised in Tables 2 and 3 respectively.

#### Clinical studies

Clinical studies with velusetrag have focused on patients with chronic constipation. The few trials that have been published have described significant improvements in bowel frequency and constipation-related symptoms vs. placebo (Supplementary Table S7).

#### Safety and tolerability

In general, there were no SAEs with velusetrag treatment; notable AEs were the predictable GI effects such as diarrhoea or altered bowel movements. In the 401-patient Phase IIb trial, 12–15% of patients developed diarrhoea on velusetrag relative to placebo (1%).^2008^ Of the three velusetrag doses tested, 15 mg provided the most favourable therapeutic index. The 50-mg dose was associated with higher prevalence of nausea, vomiting and headache than the other velusetrag doses and placebo.

An approximate 10 bpm increase in heart rate was observed following administration of 15 mg velusetrag in healthy volunteers and patients with chronic constipation.^2008^ However, in the absence of a placebo control group, this observation is difficult to interpret. The significance of the single observations of palpitations (noted with velusetrag at 30 mg) and asymptomatic junctional escape rhythm (with 50 mg dose) noted in the pharmacodynamic transit study^2010^ is uncertain, considering the limited number of subjects. In ∼540 healthy subjects or patients with chronic constipation treated with velusetrag for up to 28 days, one patient with constipation experienced palpitations in the placebo group and one healthy volunteer had junctional escape rhythm following velusetrag at 70 mg.[Bibr b135]

*In vitro* studies show that velusetrag (3 μm, 5-min application, *n* = 3 cells) had no significant effect on the magnitude of hERG potassium tail currents recorded from Chinese hamster ovary (CHO)-K1 cells expressing hERG channels.^2008^

#### Naronapride (ATI-7505)

Naronapride is structurally related to the chemical structure of cisapride, but devoid of significant affinities for other 5-HT receptors or the hERG channel.^2004^ The drug is under evaluation for treatment of upper and lower GI motility disorders, but only limited data have been published.

#### Pharmacokinetics and pharmacodynamics

The pharmacokinetics and pharmacodynamics of naronapride are summarised in Tables 2 and 3 respectively.

#### Clinical trials and indications/approvals

Naronapride has been evaluated in Phase II trials in chronic constipation, GERD and FD. Naronapride (80 mg b.d.) demonstrated significant improvement over placebo in chronic constipation.^2010^ In the upper GI tract, early Phase II studies indicated potential for reduction of reflux events and FD symptoms.[Bibr b138]

#### Safety

The cardiac safety of naronapride has been evaluated in a thorough QT study, which assesses the cardiac safety of a drug vs. both placebo and a positive control (e.g. moxifloxacin), and has confirmed a favourable safety profile at therapeutic or supratherapeutic doses.[Bibr b139]

## Discussion

Patients with symptoms that are potentially attributable to hypomotility of the GI tract constitute an important part of clinical GI practice.^2008^ These conditions are part of a spectrum that ranges from functional dysphagia and GERD, through FD and gastroparesis, to IBS-C and chronic constipation. Prokinetic drugs are considered the drugs of choice for the treatment of hypomotility disorders, although the correlation between impaired motility and symptoms is inconsistent.^2008^

The 5-HT_4_ receptor, with its location on cholinergic nerve endings of interneurons and motor neurons, has been established as a valid target to enhance GI motility. Mechanistic studies have confirmed a stimulatory effect of 5-HT_4_ receptor agonists on GI motor activity, and clinical efficacy in hypomotility disorders has been established for a number of these drugs. Cisapride, as well as tegaserod, saw broad clinical application after regulatory approval (not in the EU for the latter drug),^4^, ^5^ but both drugs were withdrawn (in the US), in part, because of cardiovascular AEs.^006^, ^007^, ^008^, ^041^ The prevalence of cardiovascular AEs with both drugs was low and likely to fall below the limit of detection in a clinical trial programme.

These events might seriously hamper the clinical development of novel 5-HT_4_ receptor agonists for the treatment of GI hypomotility disorders, especially when taking into account the ability to establish cardiovascular safety. However, several chemical classes of 5-HT_4_ receptor agonists have been developed, and the selectivity of different compounds for the 5-HT_4_ receptor over other targets is highly variable between individual drugs and drug classes. The mechanism through which cisapride promotes cardiac arrhythmias is now clearly established to be unrelated to 5-HT_4_ receptor agonism. The cardiac risk associated with cisapride use is entirely attributable to its affinity for the hERG channel which results in QT prolongation, and is enhanced by concomitant use of inhibitors of CYP, the principal pathway in cisapride metabolism.^007^, ^008^, ^041^, ^059^, ^060^, ^062^, ^063^, ^064^, ^065^, ^066^, ^067^, ^068^, ^069^ Hence, 5-HT_4_ receptor agonists without affinity for the hERG channel are devoid of this particular arrhythmogenic risk. Another risk factor in the case of cisapride was its metabolism through the CYP450 pathway, leading to increased plasma levels when other drugs also metabolised via this same pathway were taken at the same time. The mechanism through which tegaserod use may be associated with increased cardiovascular risk is not clearly established. However, it has been suggested that the affinity of tegaserod for the 5-HT_1_ or 5-HT_2(b)_ receptors may underlie these AEs.^27^, ^92^ This would implicate lack of such risk for novel, highly-selective 5-HT_4_ receptor agonists.

The link between cardiovascular risk, the CYP450 metabolic pathway and lack of selectivity of previously used 5-HT_4_ receptor agonists supports the development of selective 5-HT_4_ receptor agonists for the treatment of GI hypomotility disorders, as was the case with prucalopride for chronic constipation.^2008^ The recent EMEA approval of prucalopride for the treatment of chronic constipation confirms that 5-HT_4_ receptor agonists are still considered a valid therapeutic target at the regulatory level.^2008^ Although no link between 5-HT_4_ agonism and cardiovascular AEs has been established, standards for establishing cardiovascular safety for this class of drugs may still be elevated at the regulatory level. Indeed, the cardiovascular safety profile of prucalopride was assessed in great detail, both *in vitro* and in the clinical trial programme.^130^, ^131^, ^132^, ^038^, ^026^

Although this review may be limited by incomplete retrieval of relevant research, by bias in reporting such research, and by inherent risk of bias at the study level, our analysis of the literature has revealed a wealth of evidence that 5-HT_4_ receptor agonists have clinical efficacy in the treatment of GI disorders and no evidence of cardiovascular safety concerns with selective 5-HT_4_ receptor agonists. Nevertheless, caution is warranted as the number of patients exposed to the newer 5-HT_4_ agonists is still relatively low (number of exposed patients can be derived from the Supplementary Tables for each drug), and adequate post-marketing surveillance will help to further establish the favourable cardiovascular risk profile of these agents.

## Conclusions

5-HT_4_ receptor agonists have clear-cut prokinetic effects in the gut. These agonists differ in many aspects that are either related or unrelated to their interaction with 5-HT_4_ receptors. Differences that are unrelated to the 5-HT_4_ receptor, such as affinity at non-5-HT_4_ receptors, may influence the agent's safety and overall benefit–risk profile. 5-HT_4_ receptor-related differences have an impact on the agonists overall activity in a given tissue. Together, these differences affect the therapeutic potential for the treatment of GI motility disorders. Based on available evidence, a highly selective 5-HT_4_ receptor agonist, such as prucalopride, may offer improved efficacy and safety to treat patients with impaired GI motility, such as severe chronic constipation.

## Declaration of personal interests

Jan Tack has served as a scientific advisor for Addex pharma, AGI Therapeutics, Almirall, Aryx, AstraZeneca, Danone, Ipsen, Menarini, Movetis, Norgine, Novartis, Nycomed, Ocera, Rose Pharma, SK Life Sciences, Smartpill, Sucampo, Theravance, Tranzyme, Xenoport and Zeria, and a speaker for Abbott, AstraZeneca, Menarini, Movetis, Novartis and Nycomed. Michael Camilleri has served as a consultant and an advisory board member for Adolor, Albireo Pharma, Alkermes, Ardelyx, ARYx Therapeutics, Ascent, Astellas, AstraZeneca, BioKier, Biomedical Insights, Domain, GlaxoSmithKline, Ikaria, Ironwood, Johnson & Johnson, NovaSecta, Ocera, Procter & Gamble, Shire, SK Bio, Synergy, Takeda, Theravance and Tranzyme, and has received research funding from Albireo, NIH, Rose Pharma, Tsumura and Wyeth. Lin Chang has served as a consultant or an advisory board member for Prometheus Laboratories, Takeda, Albireo, Rose Pharma, GlaxoSmithKline, Ironwood, Forest, Salix, Marathon Pharmaceuticals, Novo Nordisk, Lexicon and Movetis, and has received research funding from Prometheus, Rose Pharma, Takeda, Ironwood and Shire. William D. Chey has served as a consultant and an advisory board member for Albireo, AstraZeneca, Forest, Ironwood, Procter & Gamble, Prometheus, Salix, Smartpill, Takeda, and has received research funding from Ardelyx and Ironwood. James J. Galligan has served as a consultant for Faegre and Benson (now Faegre, Baker, Daniels), and LLP. Brian E. Lacy has served as a speaker for Novartis, Prometheus and Takeda, and an advisory board member for Prometheus, Takeda, Ironwood and Movetis. Dr Lacy has received investigator-initiated, unrestricted funding from Takeda. Stefan Müller-Lissner has served as a speaker, a consultant and an advisory board member for Axcan, Boehringer Ingelheim, Falk Foundation, Janssen, Movetis, Menarini Farmaceutica, Movetis, Mundipharma GmbH, Novartis, Pfizer, Procter & Gamble, Sucampo Pharma and Zeria Pharma. Eamonn M. M. Quigley has served as a consultant and an advisory board member for Movetis. Dr Quigley has also served as an advisory board chair for Janssen. Jan Schuurkes is an employee of Shire. Joris H. De Maeyer is an employee of Shire. Vincenzo Stanghellini has served as speaker and advisory board member for Axcan, Danone, Janssen, Movetis, Nycomed, Norgine, Novartis, Pfizer, Sofar and Valeas. *Declaration of funding interests*: The writing or preparation of this paper was funded in part by Movetis; writing support (conducting searches and editing the manuscript) was provided by Kate Carpenter and Victoria Harvey of Choice Pharma, and was funded by Movetis.

## Supporting Information

Additional Supporting Information may be found in the online version of this article: **Table S1.** Clinical trials on the efficacy of cisapride in GI conditions. **Table S2.** Clinical trials on the efficacy of tegaserod in GI conditions. **Table S3.** Clinical trials on the efficacy of renzapride in irritable bowel syndrome. **Table S4.** Clinical trials on the efficacy of clebopride in GI conditions. **Table S5.** Clinical trials on the efficacy of mosapride in GI conditions. **Table S6.** Clinical trials on the efficacy of prucalopride in GI conditions. **Table S7.** Clinical trials on the efficacy of velusetrag in chronic constipation. Please note: Wiley-Blackwell are not responsible for the content or functionality of any supporting materials supplied by the authors. Any queries (other than missing material) should be directed to the corresponding author for the article.
