# Supplementary material for: Systematic review: cardiovascular safety profile of 5-HT4 agonists developed for gastrointestinal disorders
Source: Aliment Pharmacol Ther. 2012 Feb 22;35(7):745–67. doi: 10.1111/j.1365-2036.2012.05011.x (PMC3491670; doi:10.1111/j.1365-2036.2012.05011.x)
Supplement: Supplementary file 1 — Additional Supporting Information may be found in the online version of this article: Table S1. Clinical trials on the efficacy of cisapride in GI conditions. Table S2. Clinical trials on the efficacy of tegaserod in GI conditions. Table S3. Clinical trials on the efficacy of renzapride in irritable bowel syndrome. Table S4. Clinical trials on the efficacy of clebopride in GI conditions. Table S5. Clinical trials on the efficacy of mosapride in GI conditions. Table S6. Clinical trials on the efficacy of prucalopride in GI conditions. Table S7. Clinical trials on the efficacy of velusetrag in chronic constipation. Please note: Wiley-Blackwell are not responsible for the content or functionality of any supporting materials supplied by the authors. Any queries (other than missing material) should be directed to the corresponding author for the article. [file apt0035-0745-SD1.docx]

**Supporting information for ‘Systematic review: Cardiovascular safety profile of 5-HT_4_ agonists developed for GI disorders’**

**Table S1.** Clinical trials on the efficacy of cisapride in GI conditions

| **Reference** | **Trial design** | **Outcomes** |
| --- | --- | --- |
| **Chronic idiopathic constipation** | | |
| Bak, 1996^1^ | Prospective, OL  8 weeks  Cisapride 10 mg t.i.d. (25) | Cisapride:  Shortened total and right segmental CTT. Increased defecation frequency, and improved stool passage and consistency  Excellent global response in 46.7%, good in 33.3% and poor in 20.0% |
| Nurko, 2000^2^ | DB, PC  12 weeks  Cisapride (17) vs. placebo (19) | Cisapride vs. placebo:  Treatment success in 76% vs. 37% *(P <* 0.03)  Increased SBM/week (0.9 ± 0.1 to 4.1 ± 1.1)  Cisapride decreased:  Faecal soiling (1.8 ± 0.5 to 0.08 ± 0.4)  Encopresis in 82% vs. 23%  Number of laxative doses/week (10.3 ± 2.6 to 0.8 ± 0.6)  Percent using laxatives (from 77% to 24%)  Total GI transit time (from 115.0 ± 3.7 h to 77.0 ± 11.1 h) |
| **Gastro-oesophageal reflux disease** | | |
| Arvanitakis, 1993^3^ | R, DB  8 weeks  Cisapride 10 mg q.i.d. (18) vs*.* ranitidine 150 mg b.i.d. (19 ) | Cisapride vs. ranitidine:  Improvement of symptoms (acid regurgitation; retrosternal pain and burning; epigastric fullness and discomfort) and endoscopic healing (n.s.) |
| Baldi, 1988^4^ | DB  6–12 weeks  Cisapride 10 mg q.i.d. (21) vs. placebo (19) | Cisapride vs. placebo:  Healed lesions (no erosions, ulcers or bleeding mucosa) 63% vs. 12%, *(P* < 0.05)  Reduced antacid intake *(P* < 0.001) |
| Galmiche, 1990 ^5^ | R, DB  12 weeks  Cisapride 10 mg q.i.d. (36) vs. cimetidine 400mg q.i.d. (37) | Cisapride vs. cimetidine:  Increased mucosal healing 56% vs. 57%  Decreased intensity and frequency of heartburn; regurgitation and postural syndrome (both *P* < 0.01, n.s. between groups) |
| Geldof, 1993^6^ | DB  8–12 weeks (155)  Cisapride 10 mg q.d.s. or 20 mg b.i.d. vs. ranitidine 150 mg b.i.d. | Improvement in oesophagitis grades from BL in 68% (cisapride 10 mg), 83% (cisapride 20 mg) and 81% (ranitidine)  Endoscopically cured patients with initial grades I or II in 52% (cisapride 10 mg), 71% (cisapride 20 mg) and 80% (ranitidine) (n.s.)  Overall reflux symptom score (n.s.) |
| Schutze, 1997^7^ | R, DB, MC  8–12 weeks (407)  Cisapride 10 mg q.d.s. or 20 mg b.d.s. | Healing rates were 73% in both groups  Mean total reflux symptom score decreased from BL 7.9–2.1 (10 mg) and 7.9–2.5 (20 mg) |
| Castell, 1999^8^ | DB, PC, MC,  Cisapride 20 mg b.d.s. for 7 [±3] days (60) vs. placebo (58) | Cisapride vs. placebo:  Heartburn was prevented in 40% vs. 21%  Cisapride reduced the severity of postprandial heartburn, belching and regurgitation *(P <* 0.05) |
| Richter, 1995^9^ | R, DB  12 weeks (177)  Cisapride 10 or 20 mg q.i.d. vs. placebo | Cisapride vs.placebo:  10 mg and 20 mg reduced heartburn vs. placebo  20 mg was superior to 10 mg  Endoscopic healing in 51% (cisapride 20 mg) vs. 36% (placebo; *P* ≤ 0.044) |
| Castell, 1998^10^ | R, PC, SB  2-week run-in + 4 weeks treatment Cisapride 20 mg b.i.d. (202) or placebo (196) | Cisapride vs. placebo:  Reduced scores for heartburn *(P <* 0.001); total regurgitation (*P <* 0.001); eructation and early satiety *(P =* 0.04); antacid use (*P <* 0.001), and heartburn with antacid use (*P <* 0.001)  Increased heartburn-free days and antacid-free nights (*P <* 0.5) and patient-rated symptomatic improvement *(P=* 0.01) |
| Toussaint, 1993 ^11^ | Phase 1: OL  8–16 weeks Cisapride 10 mg q.i.d. (138)  Phase 2: R, DB  Up to 6 months  Cisapride 10 mg b.i.d. (37) vs. placebo (43) | Phase 1:  Healing in 69%  Phase 2: Cisapride vs. placebo:  Cumulative relapse rate after 6 months 21% vs. 41% *(P =* 0.06)  Global evaluation of symptoms (good or excellent) 92% vs. 69% |
| Hatlebakk, 1999 ^12^ | R, DB, PC, MC  8 weeks (483)  Cisapride 20 mg b.i.d. Omeprazole 20 mg q.d. or placebo | Adequate control of heartburn [≤1 day of the past 7 days with ≤mild heartburn in 71% (omeprazole), 22% (cisapride) and 18% (placebo)]. Omeprazole vs. cisapride and placebo *(P <* 0.0001); cisapride vs. placebo (n.s.) |
| Hatlebakk, 1997 ^13^ | R, DB, PG  Up to 6 months (535)  Cisapride 20 mg nocte or 20 mg b.i.d vs. placebo | Median time to relapse:  63 days (cisapride 20 mg b.i.d.), 59 days (cisapride 20 mg q.d.) vs. 49 days (placebo) (n.s) |
| McDougall, 1997^14^ | R  6 months (42)  Cisapride 20 mg nocte vs. placebo | Cisapride or placebo vs. BL:  Cisaparide improved lower oesophageal sphincter pressure: cisapride (*P* = 0.01), placebo (n.s.) |
| Vigneri, 1995^15^ | Phase 1:  4–8 weeks (175)  Omeprazole 40 mg q.d. until healed  Phase 2: R, DB  12 months  Cisapride 10 mg t.i.d., ranitidine 150 mg t.i.d., omeprazole 20 mg q.d., cisapride, ranitidine + cisapride 10 mg t.i.d., or omeprazole + cisapride | Remission at 12 months:  54% (cisapride), 49% (ranitidine), 80% (omeprazole), 66% (ranitidine + cisapride), 89% (omeprazole + cisapride)  Omeprazole vs. cisapride *(P =* 0.02), or vs. ranitidine *(P =* 0.003)  Omeprazole + cisapride vs. cisapride (*P =* 0.003), or vs. ranitidine *(P <* 0.001), or vs. ranitidine + cisapride (*P* = 0.03)  Ranitidine + cisapride vs. ranitidine alone (*P =* 0.05) |
| Cucchiara, 1990^16^ | Phase 1:  Cisapride 0.15 mg/Kg i.v. vs. placebo  Phase 2:  4 weeks  + oral cisapride 0.2 mg/Kg t.i.d. vs. controls (cisapride 0.15 mg/Kg i.v + dietary therapy) | Cisapride increased:  Lower oesophageal sphincter pressure (+124%), amplitude (+84%) and duration (+24%) of oesophageal peristaltic waves *(P <* 0.01). Placebo (n.s)  Cisapride vs. control decreased:  Oesophageal acid exposure in 61% vs. 24% *(P <* 0.001)  Symptom score in 61% vs. 42% *(P* < 0.01) |
| Maclennan, 2010^17^ | R  8 trials (262)  Cisapride vs. placebo or no treatment | No statistically significant difference (OR 0.34; 95% CI: 0.10–1.19) for 'same or worse' vs. 'improved symptoms' at the end of treatment  Cisapride reduced the reflux index (weighted mean difference -6.49; 95% CI: -10.13 to -2.85; *P* = 0.0005) |
| Hegar, 2009^18^ | SB, PC  1 month  (Infants regurgitating >4 times/day since >2 weeks and with reflux-associated symptoms of discomfort, after conservative treatment failure)  Cisapride vs. domperidone | Decrease in regurgitation was significant and comparable: cisapride (6.22 to 1.55) vs. domperidone (4.80 to 1.25)  Decrease in median reflux index was greater for cisapride (3.60 to 1.75) vs. domperidone (2.70 to 2.45) |
| **Functional dyspepsia** | | |
| Champion, 1997^19^ | R, DB, PG  6 weeks (123)  Cisapride 10 mg t.i.d., cisapride 20 mg t.i.d. vs. placebo | Symptom severity x frequency composite scores (n.s. between groups)  Cisapride 20 mg t.i.d. improved epigastric pain, bloating, early satiety and total symptom cluster vs. BL *(P <* 0.05) |
| De Groot, 1997^20^ | R, DB, PC  4 weeks  Cisapride 10 mg t.i.d. (56) vs. placebo (57) | Significant reduction in symptoms (n.s. between groups)  Overall response 63% (cisapride) vs. 44% (placebo, n.s.) |
| Holtmann, 1999^21^ | R, DB, DD  4 weeks (166)  Cisapride 10 mg t.i.d. vs. simethicone 84 mg t.i.d. | Simethicone vs. cisapride:  Patient assessment of symptom improvement in 46% vs. 22% *(P <* 0.01)  After 2 weeks, simethicone was better than cisapride (Δ30.7%, *P* < 0.001); after 4 weeks (Δ10.2%, *P* = 0.11) |
| Jian, 1989^22^ | R, DB  6 weeks (28)  Cisapride vs. placebo | Cisapride vs. placebo decreased:  Gastric-emptying of solids, T_1/2_= 36 ± 16 min (cisapride) vs. 227 ± 32 min (placebo, *P* < 0.02) for solids and 61 ± 4 min (cisapride) vs. 132 ± 37 min (placebo, *P* < 0.01) for liquids  Global diary score at Week 3 -16 ± 6 (cisapride) vs. -1 ± 9 (placebo, *P* < 0.05); but not at Week 6 (-18 ± 5) vs. (-10 ± 8) |
| **Diabetic gastroparesis** | | |
| Horowitz, 1987^23^ | (20)  2 days cisapride 20 mg q.d. vs. placebo, then 4 weeks of cisapride 10 mg q.i.d vs. placebo | Cisapride (20 mg) increased oesophageal emptying *(P <* 0.01) and solid and liquid gastric emptying *(P <* 0.001)  Cisapride 10 mg q.i.d. increased oesophageal emptying (*P* < 0.001) and reduced upper GI symptoms (*P* < 0.05). Placebo, n.s.)  Oesophageal emptying (n.s.) |
| Abell, 1991^24^ | 12 months  Cisapride 10 mg t.i.d.  Diabetic gastroparesis (9) vs. CIP (12) | Increased solid and liquid gastric emptying (both groups, *P* < 0.05)  Improvement of total symptom score (*P* < 0.05) in gastroparesis but not CIP |
| Braden, 2002^25^ | R  12 months  Cisapride 10 mg t.i.d. (9) vs. placebo (10) | Cisapride vs. BL:  Decreased gastric emptying (175 ± 46 min vs. 227 ± 40 min, *P* < 0.03), placebo (n.s)  Reduced dyspepsia (4.1 ± 1.6 vs. 2.0 ± 0.5, *P* = 0.002) |
| Brogna, 1989^26^ | Cisapride 10 mg i.v. (10) | Cisapride accelerated gastric emptying, placebo (n.s.) |
| Richards,1993^27^  (idiopathic and diabetic gastroparesis) | 2 week SB run-in followed by  R, DB, PC  6 weeks  Cisapride 20 mg t.i.d. (19) vs. placebo (19) | Cisapride vs BL:  Decreased gastric emptying (*P* < 0.005), placebo (n.s.) |
| Havelund, 1987 ^28^ | CO, PC (14)  Cisapride vs. placebo | Cisapride improved symptom relief and increased gastric emptying |
| Stacher, 1999^29^ | R, CO, DB  8 weeks  Cisapride 10 mg q.i.d. vs. placebo | Glycaemic control (n.s.)  Cisapride accelerated gastric emptying in 8/14 patients with delayed vs. 7/9 with non-delayed emptying (n.s.) |
| **Idiopathic gastroparesis** | | |
| Corinaldesi, 1993^30^ | 3-way, CO, DB  3x 4-day treatments separated by ≥3 days (17)  Cisapride 10 mg t.i.d for 3 days; on Day 4 (test day), 10 mg or 20 mg q.d. or 10 mg b.i.d | Greatest acceleration in gastric emptying with 10 mg b.i.d |
| **Gastroparesis and chronic intestinal pseudo-obstruction** | | |
| Camilleri, 1989^31^ | DB, PC  6 weeks  Cisapride 10 mg t.i.d. vs. placebo  (8 diabetic, 3 idiopathic gastroparesis and 15 CIP) | Cisapride vs. placebo:  Accelerated gastric emptying of solids (*P* < 0.05)  Increased postcibal antral motility and normalised abnormal manometry (tendency only)  Improvement in overall symptoms (n.s. between groups) |
| **Neuropathic chronic intestinal dysmotility** | | |
| Camilleri, 1996 ^32^ | OL 1 year  Cisapride 20 mg t.i.d. (37) | Reduced mean total symptom score vs. BL |
| Camilleri, 1994 ^33^ | R, DB, PC  12 weeks  Two-dose cisapride 10 and 20 mg t.i.d. (42) | No significant overall effect |
| **Feeding-intolerant neonates** | | |
| Enriquez, 1998 ^34^ | R, DB, PC  Cisapride 0.2 mg/Kg/dose q.i.d (18) vs. placebo (16)  Infants of ≤32 weeks of gestation | Cisapride vs. placebo:  Time taken to tolerate full enteral feeds (n.s.)  Lowered incidence of large gastric residuals and regurgitation |
| **Colonic motility in the postoperative period** | | |
| Roberts, 1995^35^ | Prospective, R, DB, PC (14)  Cisapride vs. placebo | Cisapride vs. placebo:  Increased motility index vs. placebo (*P* = 0.03) due to an increase in percentage duration of activity (*P* = 0.002) |
| **Post-operative ileus** | | |
| Lander, 1997^36^ | Prospective, R, DB  1 week  Rectal cisapride 1.4–2.3 mg/Kg/day (11) vs. placebo (12 neonates) | Cisapride vs. placebo:  First sustained feed during treatment in 73% vs. 58% at 2.3 days vs. 4.7 days |
| **Irritable bowel syndrome** | | |
| Noor, 1998^37^ | 12 weeks  Cisapride 5 mg t.i.d. (19) vs. placebo (19) | Cisapride vs. placebo:  Increased mean contraction amplitude (*P* < 0.001), duration (*P* < 0.001) and frequency (*P* < 0.001) in IBS-D, IBS-C (n.s)  Lowered symptom scores in IBS-C (*P* < 0.05)  Increased pain score in IBS-D (*P* < 0.05) |
| *b.d.s. twice a day; b.i.d., twice a day; BL, baseline; CIP, chronic intestinal pseudo-obstruction; CO, crossover; CTT, colonic transit time; DB, double-blind; DD, double dummy; GI, gastrointestinal; IBS-C, constipation-predominant IBS; IBS-D, diarrhoea-predominant IBS; i.v., intravenous; MC, multicentre; nocte, every night; n.s., not significant; OL, open-label; PC, placebo-controlled; PG, parallel group; q.d., once a day; q.d.s.,four times a day; q.i.d., four times a day; R, randomised; SB, single-blind; SBM, spontaneous bowel movements; t.i.d., three times a day* | | |

**Table S2.** Clinical trials on the efficacy of tegaserod in GI conditions

| **References** | **Study design (No. of patients)** | **Outcomes** |
| --- | --- | --- |
| **Chronic constipation** | | |
| Di Palma, 2007^38^ | R, OL, PG, MC  28 days (237)  Tegaserod 20 mg b.i.d. vs. PEG 17 g | Primary endpoint achieved in 50% of PEG and 30.8% (*P* = 0.003) of tegaserod patients (modified ROME criteria for constipation for ≥50% of their treatment weeks) |
| Nasr, 2009^39^ | OL, NCO  24 hours (22)  Tegaserod 6 mg p.o. vs. erythromycin 125 mg i.v. | Both drugs increased motor activity in antrum, duodenum and jejunum when compared to baseline period (*P* < 0.05)  The motor response with tegaserod was higher (*P* < 0.05) in jejunum and occurred during the second or third hours, whereas with erythromycin it was higher (*P* < 0.05) in antrum and occurred within 30 minutes  Following tegaserod and erythromycin, Phase III MMC occurred in 12 (55%) and 8 (36%) patients respectively (*P* > 0.05) |
| **Irritable bowel syndrome** | | |
| Ford, 2009^40^ | Meta-analysis of 11 R, PC trials  4–20 weeks  Tegaserod 0.5–12 mg b.i.d. (9242) | Therapeutic gain (RR of symptoms persisting) 10–15% (0.85; 95% CI: 0.8, 0.9)  NNT = 10 (95% CI: 8, 14) |
| Evans, 2007^41^ | Meta-analysis of 10 R or quasi-R, PC trials  Tegaserod 4 mg or 12 mg vs. placebo | Tegaserod vs. placebo:  Increased RR of being a responder (global relief of GI symptoms) during last 4 weeks of treatment  Higher responder rate in the first 4 weeks of treatment (12 mg only) vs. placebo |
| Kim, 2010^42^ | P, OL, MC  4 weeks (81 F)  Tegaserod 6 mg b.i.d. | Tegaserod reduced overall symptom scores (*P* < 0.01) and improved IBS-QoL scores (*P* < 0.01), with correlation between the two ( r = -0.60, *P* < 0.001) |
| Reitblat, 2009^43^ | Pilot study  41 F | Tegaserod lowered the IBS status from baseline 1 month after treatment (*P* < 0.001) |
| **Functional dyspepsia** | | |
| Vakil, 2008^44^ | R, DB, PC, MC  Tegaserod 6 mg b.i.d. vs. placebo (2667 F in 2 trials: [a] and [b]) | Tegaserod vs. placebo:  Mean % of days with satisfactory symptom relief:  a) 32.2% vs. 26.6% (95% CI: 2.82, 9.27; *P* < 0.01)  b) 31.9% vs. 29.4% (95% CI: -0.21, 6.53; *P* = 0.066)  Mean composite average daily severity score: a) 3.14 vs. 3.35 (95% CI: 0.29, -0.10; *P* < 0.0001)  b) 3.15 vs. 3.23 (95% CI: -0.18, 0.01; *P* = 0.094) |
| Chey, 2010^45^ | R, DB, PC  Two trials, 236, 222 days (780 F) | SF-NDI, WPAI-Dyspepsia scores and perceived symptom relief improved from baseline over the 1-year evaluation |
| *b.i.d., twice a day; DB, double-blind; F, female; GI, gastrointestinal; IBS, irritable bowel syndrome; IBS-QoL, IBS quality of life; i.v., intravenous; MC, multicentre; MMC, migrating motor complexes; NCO, non-crossover; NNT, number needed to treat; OL, open-label; PC, placebo-controlled; PEG, polyethylene glycol; PG, parallel group; p.o., per os; R, randomised; RR, relative risk; SF-NDI, Short-Form Nepean Dyspepsia Index; WPAI-Dyspepsia, Work Productivity and Activity Impairment-Dyspepsia* | | |

**Table S3.** Clinical trials on the efficacy of renzapride in irritable bowel syndrome

| **Reference** | **Study design (No. of patients)** | **Outcomes** |
| --- | --- | --- |
| **Irritable bowel syndrome** | | |
| Spiller, 2008^46^ | Phase II R, DB, PC, MC  8 weeks (168)  Renzapride 1 mg, 2 mg or 4 mg/day vs. placebo | Renzapride vs. placebo :  Patient-reported satisfactory relief of IBS symptoms (n.s.)  Responder rates (women only):  Weeks 1–4: 18.2% (95% CI: -5%, 42%; *P* = 0.066)  Weeks 5–8: 6% (95% CI: -21%, 33%; *P* = 0.339) |
| George, 2008^47^ | R, DB, PC, MC  12 weeks  Renzapride 1 mg (82), 2 mg (80) or 4 mg/day (72) vs. placebo (79) | Renzapride vs. placebo :  Patient-reported satisfactory relief of abdominal pain/discomfort (Weeks 5–12, n.s.)  Improved BM frequency and stool consistency (2 mg and 4 mg, P ≤ 0.01)  Post-hoc analysis: greater average treatment difference vs. placebo (8% vs. 12%) |
| Alizyme press release, April 2008^48^ | R  12 weeks (1821 F with IBS-C)  Renzapride 4 mg q.d. or 2 mg b.i.d. vs. placebo | Renzapride vs. placebo :  Overall symptom relief in 60% vs. 55% each week  Average improvement 5–6% vs. placebo |
| Lembo, 2010^49^ | R, DB, PC  12 weeks (1798 F)  Renzapride 4 mg q.d. or 2 mg b.i.d. vs. placebo  OL  12 months (971) | Mean (S.E.M.) number of months with relief of overall IBS symptoms was 0.55 (0.04), 0.60 (0.04) and 0.44 (0.04) with renzapride 4 mg q.d. and 2 mg b.i.d. respectively vs. placebo (P = 0.027 and P = 0.004, respectively). Renzapride produced small yet statistically significant effects on stool consistency and frequency, and bloating/abdominal distension scores |

*b.i.d., twice a day; DB, double-blind; F, female; IBS, irritable bowel syndrome; IBS-C, constipation-predominant IBS; MC, multicentre; OL, open-label; PC, placebo-controlled; q.d., once a day; R, randomised*

**Table S4.** Clinical trials on the efficacy of clebopride in GI conditions

| **Reference** | **Study design (No. of patients)** | **Outcomes** | |
| --- | --- | --- | --- |
| **Functional dyspepsia** | | | |
| Bavestrello,  1985^50^ | DB  3 months (76)  Clebopride 0.5 mg t.i.d. vs. placebo | Clebopride vs. placebo  Reduced /relieved symptoms and roentgenological findings associated with delayed gastric emptying (*P* ≤ 0.001) | |
| Sabbatini, 1991  ^51^ | R, DB  4 weeks  Clebopride 0.5 mg t.i.d. (20) or cisapride 10 mg t.i.d. (23) | Reduced dyspeptic symptoms after 2 and 4 weeks (*P* < 0.001, for both) | |
| Corinaldesi,  1985^52^ | R, DB  4 weeks (20)  Metoclopramide 10 mg t.i.d. vs. clebopride 0.5 mg t.i.d. | Metoclopramide or clebopride vs. BL:  Accelerated gastric emptying rates (*P* < 0.01)  Reduced dyspeptic symptoms (*P* < 0.05) vs. BL (metoclopramide vs. clebopride, n.s.) | |
| Nieto, 1982^53^ | CO, SB  Clebopride vs. domperidone | Clebopride vs. domperidone:  Reduced mean time for gastric evacuation, 6.75 min vs. 9.25 min (*P* < 0.01) | |
| Arienti, 1991^54^ | R, CO, DB (10)  Levosulpiride or clebopride | Levosulpiride or clebopride:  Increased gastric compliance without delaying gastric emptying (*P* < 0.05; levosulpiride vs. clebopride, n.s) | |
| **Emesis** | | | |
| Duarte, 1985^55^ | DB  (298 F undergoing elective surgery)  Clebopride 2 mg i.m. vs. placebo | | Clebopride vs. placebo: Prevented nausea (*P* ≤ 0.05) and vomiting (*P* ≤ 0.001) during the 12-hour observation period |
| *BL, baseline; CO, crossover; DB, double-blind; F, female; i.m., intramuscular; R, randomised; SB, single-blind; t.i.d., three times a day* | | | |

**Table S5.** Clinical trials on the efficacy of mosapride in GI conditions

| **Reference** | **Study design (No. of patients)** | **Outcomes** |
| --- | --- | --- |
| **Functional dyspepsia** | | |
| Amarapurkar, 2004^56^ | R, DB, PC, MC, I  2 weeks  Itopride (Ganaton) 50 mg t.i.d. (30) vs. mosapride citrate 5 mg t.i.d. (30) | Global efficacy, itopride vs. mosapride:  Patient - excellent in 17 (57%) vs. 9 (30%, *P* < 0.05) and poor in 0 (0%) vs. 3 (*P* < 0.05)  Physician - excellent in 24 (80%) vs. 15 (50%) and poor in 0 (0%) vs. 3 (10%) patients; excellent to good in 93.3% vs. mosapride 63.3% (*P* < 0.05)  2 patients (6.7%) on mosapride withdrew due to AEs |
| Kinoshita, 2005^57^ | R, MC  4 weeks  Famotidine vs. mosapride vs. tandospirone | Symptom relief (VAS):  Famotidine > mosapride > tandospirone  Famotidine >> tandospirone |
| Otaka, 2005^58^ | R  4 weeks  Mosapride 15 mg/day vs. famotidine 20 mg/day [Non-responders followed on with amitriptyline 30 mg/day vs. no medication for 4 weeks] | Symptom relief (VAS):  Mosapride = famotidine |
| Seno, 2005^59^ | R  8 weeks (62)  Mosapride 15 mg/day vs. famotidine 40 mg/day vs. tandospirone 30 mg/day | Symptom relief (VAS) vs. BL:  Mosapride (1.57 ± 0.20 vs. 2.29 ± 0.14); famotidine (1.09 ± 0.12 vs. 2.0 ± 0.16 (at 2 weeks, *P* < 0.01); Famotidine > mosapride (*P* < 0.05); tandospirone (n.s.) |
| **Non-erosive reflux disease** | | |
| Futagami, 2009^60^ | 12 weeks (44 PPI-resistant NERD patients, 20 HV)  Combination therapy: mosapride citrate 15 mg/day + omeprazole 20 mg/day | Significantly improved reflux symptoms and T_max_ value in T_max_ >65 min NERD patients  Significantly reduced des-acylated-ghrelin levels in NERD patients with delayed gastric emptying |
| *AE, adverse event; BL, baseline; DB, double-blind; GI, gastrointestinal; HV, healthy volunteers; I, international; MC, multicentre; NERD, non-erosive reflux disease; n.s., not significant; PC, placebo-controlled; PPI, proton pump inhibitor; R, randomised; VAS, visual analogue scale* | | |

**Table S6.** Clinical trials on the efficacy of prucalopride in GI conditions

| **Reference** | **Study design (No. of patients)** | **Outcomes** |
| --- | --- | --- |
| **Constipation** | | |
| Camilleri, 2008 ^61^ | R, PC, PG, MC  12 weeks (620)  Prucalopride 2 mg or 4 mg (q.d.) vs. placebo | Prucalopride 2 mg and 4 mg vs. placebo:  Increased no. of patients achieving:  ≥3 SCBM/week in 30.9% and 28.4% vs. 12% (*P* < 0.001)  An increase of ≥1 SCBM/week in 47.3% and 46.6% vs. 25.8% (*P* < 0.001) |
| Emmanuel, 2002 ^62^ | R, DB, PC  4 weeks (74 women)  Prucalopride 1 mg (q.d.) vs. placebo | Prucalopride (but not placebo) increased SBM frequency (*P* = 0.008) and reduced time to first stool (*P* < 0.001), accelerated orocaecal transit (*P* = 0.004), increased rectal sensitivity (*P* ≤ 0.01), improved several domains of the Short Form Health Status Survey and the disease-specific QoL  Prucalopride vs. placebo reduced the no. of retained markers in all patients (*P* = 0.004); reduced the mean no. of retained markers in STC (*P* = 0.069), but did not alter the marker count in NTC (*P* = 0.86) |
| Krogh, 2002 ^63^ | R, DB, PC, pilot  4 weeks  4 weeks run-in then prucalopride 1 mg (8) or placebo (4); for new patients, prucalopride 2 mg (q.d.) (8) vs. placebo (3) | Dose-related mean decreases in constipation severity (VAS) and increases with placebo  Increased BM frequency (2 mg, median 0.6; 95% CI: 0.2; 1.2)  Significant reduction in median CTT with 2 mg (*n* = 4; -38.5 H [95% CI: -80, -5]) |
| Quigley, 2009 ^64^ | R, DB, PC  12 weeks (641)  Prucalopride 2 or 4 mg (q.d.) vs. placebo | Prucalopride 2 mg and 4 mg vs. placebo:  Increased no. of patients achieving:  ≥3 SCBM/week in prucalopride 2 or 4 mg (24%) vs. placebo (12%)  An increase of ≥1 SCBM/week in 43% and 47% vs. 28%, respectively |
| Tack, 2009 ^65^ | R, PC, PG, MC  12 weeks (713)  Prucalopride 2 or 4 mg (q.d.) vs. placebo | Prucalopride 2 mg and 4 mg vs. placebo:  Increased no. of patients achieving:  ≥3 SCBM/week in 19.5% (*P* < 0.01) and 23.6% (*P* < 0.001) vs. 9.6%  An increase of ≥1 SCBM/week, evacuation completeness, perceived disease severity and treatment effectiveness and QOL  Prucalopride 4 mg reduced the need for straining vs. placebo (*P* < 0.05) |
| Müller-Lissner, 2010 ^66^ | R, DB, PC  4 weeks (300 aged ≥65 years)  Prucalopride 1, 2 or 4 mg (q.d.) vs. placebo | More patients achieved ≥3 SCBM/week with prucalopride vs. placebo (*P* ≤ 0.05 in the 1^st^ week of 4 mg prucalopride)  More patients achieved an increase of ≥1 SCBM/week from baseline: 1 mg prucalopride (60%) vs. placebo (34%) at week 4 (*P* ≤ 0.05)  More patients had improvement in PAC-QOL satisfaction score of ≥1 with 1 mg prucalopride vs. placebo (*P* ≤ 0.05) and in PAC-SYM stool symptoms (1 and 4 mg prucalopride; *P* ≤ 0.05) |
| Sloots, 2010 ^67^ | R, DB, PC  4 weeks  Prucalopride 2 mg (66) or 4 mg (64) vs. placebo (66) | More patients had an increase from baseline of ≥1 SCBM/week with prucalopride 2 mg (35.9%) and 4 mg (40.3%) vs. placebo (23.4%), reaching statistical significance in Week 1  More patients achieved an average of ≥3 SBM/week with prucalopride 2 mg (60.7%) and 4 mg (69%) vs. placebo (43.3%), reaching statistical significance at Week 1  Prucalopride 4 mg significantly improved patient-rated severity of constipation and effectiveness of treatment vs. placebo  PAC-SYM total scores and PAC-QoL total and satisfaction subscale scores were improved |
| Dubois, 2010 ^68^ | R, DB, PC  3 trials, 12 weeks  Prucalopride (q.d.) | Prucalopride vs. placebo showed superior effects on PAC-QOL scores |
| Frampton, 2009 ^69^ | R, DB, PC, MC  12 weeks  Prucalopride 2 mg or 4 mg (q.d.) vs. placebo | Prucalopride vs. placebo significantly improved patient-assessed bowel habits, constipation symptoms and severity, satisfaction with bowel habit and treatment, and HRQoL  Maintained for up to 24 months in OL long-term follow-up studies |
| **Scleroderma** | | |
| Boeckxstaens, 2002 ^70^ | Pilot  Prucalopride | Symptomatic improvement and increased BM frequency in patients in whom previous prokinetic treatment had failed |
| *CTT, colonic transit time; DB, double-blind; MC, multicentre; NTC, normal transit constipation; OL, open-label; PAC-QoL, Patient Assessment of Constipation-Quality of Life; PAC-SYM, Patient Assessment of Constipation-Symptom; PC, placebo-controlled; PG, parallel group; q.d., once a day; QoL, quality of life; R, randomised; SBM, spontaneous bowel movements; SCBM, spontaneous complete bowel movements; VAS, visual analogue scale* | | |

**Table S7.** Clinical trials on the efficacy of velusetrag in chronic constipation

| **Reference** | **Study design (No. of patients)** | **Outcomes** |
| --- | --- | --- |
| **Chronic constipation** |  |  |
| Manini, 2010 ^71^ | R, DB, PC  (60 healthy volunteers)  5, 15, 30 or 50 mg TD5108 (single and 6-day dosing) vs. placebo | Bowel function after 15 mg TD5108 was similar in controls and chronic constipation patients |
| Goldberg, 2008 ^72^ | R, DB, PC, PG, MC  4 weeks  TD5108 15 mg (95), 30 mg (91) or 50 mg (84) q.d. vs. placebo (98) | TD5108 15 mg, 30 mg or 50 mg vs. placebo  Increase of ≥1 SCBM/week in 42% (*P* < 0.001), 30% (*P* = 0.01) and 39% (*P* < 0.001) vs. 13%, respectively  ≥3 SCBM/week in 27% (*P* < 0.001), 19% (*P* = 0.006) and 21% (*P* = 0.002) vs. 5%, respectively |
| *DB, double-blind; PC, placebo-controlled; PG, parallel group; q.d., once a day; R, randomised; SCBM, spontaneous complete bowel movements* | | |

**References**

1. Bak YT, Kim JH, Lee CH. Cisapride in chronic idiopathic constipation: clinical response and effect on colonic transit time. *Korean J Intern Med* 1996; **11**: 151–6.

2. Nurko S, Garcia-Aranda JA, Worona LB, Zlochisty O. Cisapride for the treatment of constipation in children: A double-blind study. *J Pediatr* 2000; **136**: 35–40.

3. Arvanitakis C, Nikopoulos A, Theoharidis A*, et al.* Cisapride and ranitidine in the treatment of gastro-oesophageal reflux disease--a comparative randomized double-blind trial. *Aliment Pharmacol Ther* 1993; **7**: 635–41.

4. Baldi F, Bianchi Porro G, Dobrilla G*, et al.* Cisapride versus placebo in reflux esophagitis. A multicenter double-blind trial. *J Clin Gastroenterol* 1988; **10**: 614–8.

5. Galmiche JP, Fraitag B, Filoche B*, et al.* Double-blind comparison of cisapride and cimetidine in treatment of reflux esophagitis. *Dig Dis Sci* 1990; **35**: 649–55.

6. Geldof H, Hazelhoff B, Otten MH. Two different dose regimens of cisapride in the treatment of reflux oesophagitis: a double-blind comparison with ranitidine. *Aliment Pharmacol Ther* 1993; **7**: 409–15.

7. Schutze K, Bigard MA, Van Waes L, Hinojosa J, Bedogni G, Hentschel E. Comparison of two dosing regimens of cisapride in the treatment of reflux oesophagitis. *Aliment Pharmacol Ther* 1997; **11**: 497–503.

8. Castell D, Silvers D, Littlejohn T*, et al.* Cisapride 20 mg b.d. for preventing symptoms of GERD induced by a provocative meal. The CIS-USA-89 Study Group. *Aliment Pharmacol Ther* 1999; **13**: 787–94.

9. Richter JE, Long JF. Cisapride for gastroesophageal reflux disease: a placebo-controlled, double-blind study. *Am J Gastroenterol* 1995; **90**: 423–30.

10. Castell DO, Sigmund C, Jr., Patterson D*, et al.* Cisapride 20 mg b.i.d. provides symptomatic relief of heartburn and related symptoms of chronic mild to moderate gastroesophageal reflux disease. CIS-USA-52 Investigator Group. *Am J Gastroenterol* 1998; **93**: 547–52.

11. Toussaint J, Gossuin A, Deruyttere M, Huble F, Devis G. Healing and prevention of relapse of reflux oesophagitis by cisapride. *Gut* 1991; **32**: 1280–5.

12. Hatlebakk JG, Hyggen A, Madsen PH*, et al.* Heartburn treatment in primary care: randomised, double blind study for 8 weeks. *BMJ* 1999; **319**: 550–3.

13. Hatlebakk JG, Johnsson F, Vilien M, Carling L, Wetterhus S, Thogersen T. The effect of cisapride in maintaining symptomatic remission in patients with gastro-oesophageal reflux disease. *Scand J Gastroenterol* 1997; **32**: 1100–6.

14. McDougall NI, Watson RG, Collins JS, McFarland RJ, Love AH. Maintenance therapy with cisapride after healing of erosive oesophagitis: a double-blind placebo-controlled trial. *Aliment Pharmacol Ther* 1997; **11**: 487–95.

15. Vigneri S, Termini R, Leandro G*, et al.* A comparison of five maintenance therapies for reflux esophagitis. *N Engl J Med* 1995; **333**: 1106–10.

16. Cucchiara S, Staiano A, Boccieri A*, et al.* Effects of cisapride on parameters of oesophageal motility and on the prolonged intraoesophageal pH test in infants with gastro-oesophageal reflux disease. *Gut* 1990; **31**: 21–5.

17. Maclennan S, Augood C, Cash-Gibson L, Logan S, Gilbert RE. Cisapride treatment for gastro-oesophageal reflux in children. *Cochrane Database Syst Rev* 2010; **4**: CD002300.

18. Hegar B, Alatas S, Advani N, Firmansyah A, Vandenplas Y. Domperidone versus cisapride in the treatment of infant regurgitation and increased acid gastro-oesophageal reflux: a pilot study. *Acta Paediatr* 2009; **98**: 750–5.

19. Champion MC, MacCannell KL, Thomson AB*, et al.* A double-blind randomized study of cisapride in the treatment of nonulcer dyspepsia. The Canadian Cisapride Nud Study Group. *Can J Gastroenterol* 1997; **11**: 127–34.

20. de Groot GH, de Both PS. Cisapride in functional dyspepsia in general practice. A placebo-controlled, randomized, double-blind study. *Aliment Pharmacol Ther* 1997; **11**: 193–9.

21. Holtmann G, Gschossmann J, Karaus M*, et al.* Randomised double-blind comparison of simethicone with cisapride in functional dyspepsia. *Aliment Pharmacol Ther* 1999; **13**: 1459–65.

22. Jian R, Ducrot F, Ruskone A*, et al.* Symptomatic, radionuclide and therapeutic assessment of chronic idiopathic dyspepsia. A double-blind placebo-controlled evaluation of cisapride. *Dig Dis Sci* 1989; **34**: 657–64.

23. Horowitz M, Maddox A, Harding PE*, et al.* Effect of cisapride on gastric and esophageal emptying in insulin-dependent diabetes mellitus. *Gastroenterology* 1987; **92**: 1899–907.

24. Abell TL, Camilleri M, DiMagno EP, Hench VS, Zinsmeister AR, Malagelada JR. Long-term efficacy of oral cisapride in symptomatic upper gut dysmotility. *Dig Dis Sci* 1991; **36**: 616–20.

25. Braden B, Enghofer M, Schaub M, Usadel KH, Caspary WF, Lembcke B. Long-term cisapride treatment improves diabetic gastroparesis but not glycaemic control. *Aliment Pharmacol Ther* 2002; **16**: 1341–6.

26. Brogna A, Ferrara R, Scornavacca G*, et al.* Cisapride and gastric emptying of a solid meal in dyspeptic diabetics without autonomic neuropathy and in healthy volunteers. *Eur J Clin Pharmacol* 1989; **37**: 411–3.

27. Richards RD, Valenzuela GA, Davenport KG, Fisher KL, McCallum RW. Objective and subjective results of a randomized, double-blind, placebo-controlled trial using cisapride to treat gastroparesis. *Dig Dis Sci* 1993; **38**: 811–6.

28. Havelund T, Oster-Jorgensen E, Eshoj O, Larsen ML, Lauritsen K. Effects of cisapride on gastroparesis in patients with insulin-dependent diabetes mellitus. A double-blind controlled trial. *Acta Med Scand* 1987; **222**: 339–43.

29. Stacher G, Schernthaner G, Francesconi M*, et al.* Cisapride versus placebo for 8 weeks on glycemic control and gastric emptying in insulin-dependent diabetes: a double blind cross-over trial. *J Clin Endocrinol Metab* 1999; **84**: 2357–62.

30. Corinaldesi R, Stanghellini V, Tosetti C*, et al.* The effect of different dosage schedules of cisapride on gastric emptying in idiopathic gastroparesis. *Eur J Clin Pharmacol* 1993; **44**: 429–32.

31. Camilleri M, Malagelada JR, Abell TL, Brown ML, Hench V, Zinsmeister AR. Effect of six weeks of treatment with cisapride in gastroparesis and intestinal pseudoobstruction. *Gastroenterology* 1989; **96**: 704–12.

32. Camilleri M, Balm RK, Zinsmeister AR. Symptomatic improvement with one-year cisapride treatment in neuropathic chronic intestinal dysmotility. *Aliment Pharmacol Ther* 1996; **10**: 403–9.

33. Camilleri M, Balm RK, Zinsmeister AR. Determinants of response to a prokinetic agent in neuropathic chronic intestinal motility disorder. *Gastroenterology* 1994; **106**: 916–23.

34. Enriquez A, Bolisetty S, Patole S, Garvey PA, Campbell PJ. Randomised controlled trial of cisapride in feed intolerance in preterm infants. *Arch Dis Child Fetal Neonatal Ed* 1998; **79**: F110–3.

35. Roberts JP, Benson MJ, Rogers J, Deeks JJ, Wingate DL, Williams NS. Effect of cisapride on distal colonic motility in the early postoperative period following left colonic anastomosis. *Dis Colon Rectum* 1995; **38**: 139–45.

36. Lander A, Redkar R, Nicholls G*, et al.* Cisapride reduces neonatal postoperative ileus: randomised placebo controlled trial. *Arch Dis Child Fetal Neonatal Ed* 1997; **77**: F119–22.

37. Noor N, Small PK, Loudon MA, Hau C, Campbell FC. Effects of cisapride on symptoms and postcibal small-bowel motor function in patients with irritable bowel syndrome. *Scand J Gastroenterol* 1998; **33**: 605–11.

38. Di Palma JA, Cleveland MV, McGowan J, Herrera JL. A randomized, multicenter comparison of polyethylene glycol laxative and tegaserod in treatment of patients with chronic constipation. *Am J Gastroenterol* 2007; **102**: 1964–71.

39. Nasr I, Rao SS, Attaluri A, Hashmi SM, Summers R. Effects of tegaserod and erythromycin in upper gut dysmotility: a comparative study. *Indian J Gastroenterol* 2009; **28**: 136–42.

40. Ford AC, Brandt LJ, Young C, Chey WD, Foxx-Orenstein AE, Moayyedi P. Efficacy of 5-HT(3) Antagonists and 5-HT(4) Agonists in Irritable Bowel Syndrome: Systematic Review and Meta-Analysis. *Am J Gastroenterol* 2009; **104**: 1831–43, quiz 44.

41. Evans BW, Clark WK, Moore DJ, Whorwell PJ. Tegaserod for the treatment of irritable bowel syndrome and chronic constipation. *Cochrane Database Syst Rev* 2007: CD003960.

42. Kim YS, Choi SC, Park JM*, et al.* The effect of tegaserod on symptoms and quality of life in korean women with irritable bowel syndrome with constipation. *J Neurogastroenterol Motil* 2010; **16**: 61–70.

43. Reitblat T, Zamir D, Polishchuck I, Novochatko G, Malnick S, Kalichman L. Patients treated by tegaserod for irritable bowel syndrome with constipation showed significant improvement in fibromyalgia symptoms. A pilot study. *Clin Rheumatol* 2009; **28**: 1079–82.

44. Vakil N, Laine L, Talley NJ*, et al.* Tegaserod treatment for dysmotility-like functional dyspepsia: results of two randomized, controlled trials. *Am J Gastroenterol* 2008; **103**: 1906–19.

45. Chey WD, Howden CW, Tack J, Ligozio G, Earnest DL. Long-term tegaserod treatment for dysmotility-like functional dyspepsia: results of two identical 1-year cohort studies. *Dig Dis Sci* 2010; **55**: 684–97.

46. Spiller RC, Meyers NL, Hickling RI. Identification of patients with non-d, non-C irritable bowel syndrome and treatment with renzapride: an exploratory, multicenter, randomized, double-blind, placebo-controlled clinical trial. *Dig Dis Sci* 2008; **53**: 3191–200.

47. George AM, Meyers NL, Hickling RI. Clinical trial: renzapride therapy for constipation-predominant irritable bowel syndrome--multicentre, randomized, placebo-controlled, double-blind study in primary healthcare setting. *Aliment Pharmacol Ther* 2008; **27**: 830–7.

48. Alizyme. Press release: Results from Renzapride. April 2008. Available from: <http://www.alizyme.com/alizyme/media/press/show.jsp?ref=128>.

49. Lembo AJ, Cremonini F, Meyers N, Hickling R. Clinical trial: renzapride treatment of women with irritable bowel syndrome and constipation - a double-blind, randomized, placebo-controlled, study. *Aliment Pharmacol Ther* 2010; **31**: 979–90.

50. Bavestrello L, Caimi L, Barbera A. A double-blind comparison of clebopride and placebo in dyspepsia secondary to delayed gastric emptying. *Clin Ther* 1985; **7**: 468–73.

51. Sabbatini F, Minieri M, Manzi G, Piai G, D'Angelo V, Mazzacca G. Clinical efficacy and safety of cisapride and clebopride in the management of chronic functional dyspepsia: a double-blind, randomized study. *Ital J Gastroenterol* 1991; **23**: 1–4.

52. Corinaldesi R, Stanghellini V, Raiti C, Zarabini E, Rea E, Paparo GF. Effects of chronic oral administration of clebopride and metoclopramide on gastric emptying of solids in patients with functional dyspepsia. *Curr Ther Res* 1985; **38**: 790–7.

53. Nieto CM. Effects of chronic oral administration of clebopride and metoclopramide on gastric emptying of solids in patients with functional dyspepsia *Curr Ther Res* 1982; **31**: 69–73.

54. Arienti V, Magri F, Boriani L, Belotti M, Ugenti F, Gasbarrini G. Levosulpiride versus clebopride in gastric and gallbladder emptying in patients with functional dyspepsia: Ultrasonographic evaluation. *Curr Ther Res* 1991; **49**: 575–87.

55. Duarte DF, Linhares S, Gesser N, Pederneiras SG. Effect of intramuscular clebopride on postoperative nausea and vomiting. *Clin Ther* 1985; **7**: 365–71.

56. Amarapurkar DN, Rane P. Randomised, double-blind, comparative study to evaluate the efficacy and safety of ganaton (itopride hydrochloride) and mosapride citrate in the management of functional dyspepsia. *J Indian Med Assoc* 2004; **102**: 735–7, 60.

57. Kinoshita Y, Hashimoto T, Kawamura A*, et al.* Effects of famotidine, mosapride and tandospirone for treatment of functional dyspepsia. *Aliment Pharmacol Ther* 2005; **21 Suppl 2**: 37–41.

58. Otaka M, Jin M, Odashima M*, et al.* New strategy of therapy for functional dyspepsia using famotidine, mosapride and amitriptyline. *Aliment Pharmacol Ther* 2005; **21 Suppl 2**: 42–6.

59. Seno H, Nakase H, Chiba T. Usefulness of famotidine in functional dyspepsia patient treatment: comparison among prokinetic, acid suppression and antianxiety therapies. *Aliment Pharmacol Ther* 2005; **21 Suppl 2**: 32–6.

60. Futagami S, Iwakiri K, Shindo T*, et al.* The prokinetic effect of mosapride citrate combined with omeprazole therapy improves clinical symptoms and gastric emptying in PPI-resistant NERD patients with delayed gastric emptying. *J Gastroenterol* 2010; **45**: 413–21.

61. Camilleri M, Kerstens R, Rykx A, Vandeplassche L. A placebo-controlled trial of prucalopride for severe chronic constipation. *N Engl J Med* 2008; **358**: 2344–54.

62. Emmanuel AV, Roy AJ, Nicholls TJ, Kamm MA. Prucalopride, a systemic enterokinetic, for the treatment of constipation. *Aliment Pharmacol Ther* 2002; **16**: 1347–56.

63. Krogh K, Jensen MB, Gandrup P*, et al.* Efficacy and tolerability of prucalopride in patients with constipation due to spinal cord injury. *Scand J Gastroenterol* 2002; **37**: 431–6.

64. Quigley EM, Vandeplassche L, Kerstens R, Ausma J. Clinical trial: the efficacy, impact on quality of life, and safety and tolerability of prucalopride in severe chronic constipation--a 12-week, randomized, double-blind, placebo-controlled study. *Aliment Pharmacol Ther* 2009; **29**: 315–28.

65. Tack J, van Outryve M, Beyens G, Kerstens R, Vandeplassche L. Prucalopride (Resolor) in the treatment of severe chronic constipation in patients dissatisfied with laxatives. *Gut* 2009; **58**: 357–65.

66. Muller-Lissner S, Rykx A, Kerstens R, Vandeplassche L. A double-blind, placebo-controlled study of prucalopride in elderly patients with chronic constipation. *Neurogastroenterol Motil* 2010; **22**: 991–8.

67. Sloots CE, Rykx A, Cools M, Kerstens R, De Pauw M. Efficacy and Safety of Prucalopride in Patients with Chronic Noncancer Pain Suffering from Opioid-Induced Constipation. *Dig Dis Sci* 2010; **55**: 2912–21.

68. Dubois D, Gilet H, Viala-Danten M, Tack J. Psychometric performance and clinical meaningfulness of the Patient Assessment of Constipation-Quality of Life questionnaire in prucalopride (RESOLOR) trials for chronic constipation. *Neurogastroenterol Motil* 2010; **22**: e54–63.

69. Frampton JE. Prucalopride. *Drugs* 2009; **69**: 2463–76.

70. Boeckxstaens GE, Bartelsman JF, Lauwers L, Tytgat GN. Treatment of GI dysmotility in scleroderma with the new enterokinetic agent prucalopride. *Am J Gastroenterol* 2002; **97**: 194–7.

71. Manini ML, Camilleri M, Goldberg M*, et al.* Effects of Velusetrag (TD-5108) on gastrointestinal transit and bowel function in health and pharmacokinetics in health and constipation. *Neurogastroenterol Motil* 2010; **22**: 42–9, e7–8.

72. Goldberg MR, Li YP, Pitzer K, Johanson JF, Mangel A, Kitt MM. In patients with chronic constipation, TD-5108, a selective 5-HT4 agonist with high intrinsic activity, relieves straining and bloating, normalizes stool consistency and reduces laxative use. *Gastroenterology* 2008; **134** (abstract T1389).
